# Cross-regulation of Aps-promoters in *Lacticaseibacillus paracasei* by the PsdR response regulator in response to lantibiotics

**DOI:** 10.1038/s41598-024-53592-1

**Published:** 2024-02-09

**Authors:** Qian Zhang, Manuel Zúñiga, Cristina Alcántara, Diana Wolf, Thorsten Mascher, Ainhoa Revilla-Guarinos

**Affiliations:** 1 Chair of General Microbiology, Technische Universität Dresden, 01217 Dresden, Germany; 2 Departamento de Biotecnología, Instituto de Agroquímica y Tecnología de Alimentos (IATA), Consejo Superior de Investigaciones Científicas (CSIC), 46980 Paterna, Valencia Spain; 3 Present Address: Oral Microbiome Group, Genomics and Health Department, FISABIO Foundation, 46020 Valencia, Spain

**Keywords:** Antimicrobial resistance, Food microbiology, Peptides, Gene expression profiling

## Abstract

The PsdRSAB and ApsRSAB detoxification modules, together with the antimicrobial peptides (AMPs)-resistance determinants Dlt system and MprF protein, play major roles in the response to AMPs in *Lacticaseibacillus paracasei* BL23. Sensitivity assays with a collection of mutants showed that the PsdAB ABC transporter and the Dlt system are the main subtilin resistance determinants. Quantification of the transcriptional response to subtilin indicate that this response is exclusively regulated by the two paralogous systems PsdRSAB and ApsRSAB. Remarkably, a cross-regulation of the *derAB*, *mprF* and *dlt-*operon genes—usually under control of ApsR—by PsdR in response to subtilin was unveiled. The high similarity of the predicted structures of both response regulators (RR), and of the RR-binding sites support this possibility, which we experimentally verified by protein-DNA binding studies. ApsR-P shows a preferential binding in the order P_*derA*_ > P_*dlt*_ > P_*mprF*_ > P_*psdA*_. However, PsdR-P bound with similar apparent affinity constants to the four promoters. This supports the cross-regulation of *derAB*, *mprF* and the *dlt-*operon by PsdR. The possibility of cross-regulation at the level of RR-promoter interaction allows some regulatory overlap with two RRs controlling the expression of systems involved in maintenance of critical cell membrane functions in response to lantibiotics.

## Introduction

Bacteria are often exposed to changing and challenging environmental conditions. This necessitates communication systems that perceive and process information from their direct surroundings and initiate appropriate cellular responses. Bacterial Two-Component Systems (TCS) are sophisticated signal transduction devices that specifically connect external stimulus with appropriate cellular responses ensuring the microorganism survival^1^. Consequently, microorganisms inhabiting rapidly changing environments usually possess a higher number of TCS than those inhabiting more stable niches^2^.

TCS typically consists of two proteins, a Histidine Kinase (HK) and a Response Regulator (RR), both containing a modular domain structure. HKs typically contain an N-terminal sensing domain, which is often located in the extracellular space, and a C-terminal cytoplasmic transmitter domain. The former perceives the environmental stimulus, while in response the latter autophosphorylates at a conserved histidine residue. RRs consist of an N-terminal receiver domain, which is phosphorylated by the cognate HK at a conserved aspartyl residue, and a C-terminal effector (usually DNA-binding) domain. This phosphorylation results in a conformational change that modulates the activity of the effector domain to act as a transcriptional activator/repressor^3^.

HKs and RRs each comprise paralogous gene families and the members of each family share significant homology at both the primary sequence and structural level^4^. A remarkable characteristic of TCS is the specificity of the interactions between partner HKs and RRs despite the high conservation of the transmitter and receiver domains. This specificity of the phosphotransfer reactions is ensured by molecular and structural recognition mechanisms^5^. This characteristic is important as a way to ensure the activation of the necessary response to a specific stimulus. However, under some conditions, cross-regulation integrating different signals or diversifying the response to a single stimulus may be advantageous^4^.

So-called Bce-like systems, named after the BceRSAB system from *Bacillus subtilis*, represent unique variations of TCS-dependent signal transduction^6,7^. They differ from canonical TCS in two aspects: (i) They contain intramembrane-sensing BceS-like HKs, which lack an extracellular sensing domain and do not function as sensors of the system^8^. (ii) BceRS-like TCS appear genetically and functionally associated with the BceAB-like ABC transporters which can act simultaneously as antimicrobial peptide (AMPs) sensors and resistance detoxification pumps^9–12^. The resistance mechanism involves the transfer of the signal from the ABC transporter to its cognate HK by modifying its conformational state^13,14^. Subsequent phosphorylation of the associated RR induces the expression of the resistance genes. By this mechanism, the cell is able to adjust the amount of the AMP transporters in the membrane to the need posed by the current AMP threat from the outside^12^.

The probiotic Gram-positive bacterium *Lacticaseibacillus paracasei* BL23 possesses a total number of 17 TCS^15,16^. Two of them, PsdRS and ApsRS are homologous to BceRS from *B. subtilis*^17^. They are genomically and functionally associated with the BceAB-like transporters PsdAB and ApsAB, respectively^17^. TCSs and cognate ABC transporters constitute functional units (initially referred to as module 09 and module 12^17^). PsdRSAB is a stand-alone detoxification system with PsdAB acting as both a sensing and resistance transporter: PsdRS responds to nisin via PsdAB, whose expression is induced by PsdRS thereby conferring nisin resistance. PsdRSAB additionally confers resistance against bacitracin, plectasin, and subtilin^17^. In contrast, ApsRSAB only contains a sensory transporter not directly involved in AMPs resistance. Instead, this system regulates the expression of a larger regulon that includes the *dlt* operon, the *mprF* gene, and the defensin-specific, BceAB-like resistance transporter *derAB*^18^. The *dltABCD* operon encodes the four proteins of the DltABCD system, which catalyzes the D-alanylation of teichoic acids (TAs) in *Firmicutes* bacteria^19^. The degree of D-alanylation of TAs has a wide range of physiological effects^19–21^, among them, the resistance against cationic antimicrobial peptides (CAMPs). It is postulated that D-alanylation reduces the net negative charge of the cell envelope and increases the peptidoglycan sacculus density, thus decreasing electrostatic interactions with CAMPs and impairing their passage across the cell wall (reviewed in^12^). *mprF* encodes the MprF protein, which catalyzes the lysinylation of membrane phospholipids^22,23^. The activity of MprF also plays a role in AMPs resistance by reducing the access to their molecular targets on the cytoplasmic membrane^24–27^. ApsRSAB deletion mutants show a decreased expression of the Dlt system and DerAB. Consequently, ApsRSAB deletion mutants are sensitive to insect defensins, to the bacteriocins bacitracin, nisin, subtilin, mersacidin and vancomycin, to the fungal defensin plectasin, and the human cathelicin LL37. The decreased expression of the Dlt system also renders these mutants acid sensitive^28^.

Interestingly, our previous studies^18^ suggested that the Bce-like systems from *L. paracasei* BL23 have still not fully diverged^29^, that is, are still in the process of achieving the complete regulatory insulation from one another: We showed that even though the mutants in the ApsRSAB system had a much lower level of expression of the *dlt* operon and the *mprF* gene compared to that of the wild type, a minor nisin-dependent induction of both was still observed. These result suggested that additional regulatory systems might be able to control *dlt*/mprF expression in the absence of a functional ApsRSAB module^17^. Later, we showed that spurious interactions of DerB with noncognate protein partners from the Psd system might result in a negative impact on PsdRSAB signal transduction. This idea was supported by the results showing that a *derB* deletion allowed the full potential activation of the PsdRSAB response to nisin as reflected by an increased expression of the *psdAB* transporter and a greater level of resistance against nisin^18^. Interestingly, removing *derB* also resulted in an increased induction of the *derA*, *dltA* and *mprF* genes, which are regulated by the Aps system. The residual induction of the Aps-regulated genes in the absence of a functional Aps system^17^, and the increased induction of those same genes by nisin—but not subtilin—in ∆*derB*^18^ raised the question of a possible cross-regulation of the Aps regulon by the Psd system.

In this report we investigated the response of *L. paracasei* BL23 to subtilin in detail. Our results indicate that the Psd system might indeed cross-regulate the Aps-target genes, involved in maintenance of critical cell membrane functions, at least in the absence of ApsRSAB.

## Results

### Psd and Aps regulons mediate the response of *L. paracasei* BL23 to subtilin

In a previous study, we described a hierarchical regulatory network mediating resistance against insect-derived antimicrobial peptides in *L. paracasei* BL23. In this network, the ApsRSAB system represents the primary regulator, which controls all the genes encoding the Dlt system, the MprF protein and the DerAB transporter^18^. We wondered if a similar hierarchical response could mediate subtilin resistance in BL23. To investigate this hypothesis, a set of mutants in Bce-like modules and the genes under their regulation was created (Supplementary Table S1) and their sensitivity against subtilin was determined. In most cases, exposition to subinhibitory concentrations did not affect the final OD reached by the cultures after 20–24 h but it lengthened the lag phase of the cultures (see exemplary graphs in Supplementary Fig. S1). Taking this effect in consideration, the sensitivity of the mutant strains against subtilin was determined as the Minimal Inhibitory Concentration after 15 h (MIC_15H_) of exposure to the AMP, since differences in cellular growth at 15 h reflect better the differential response of the mutant strains to subinhibitory concentrations of the AMPs under study.

**Figure 1:**
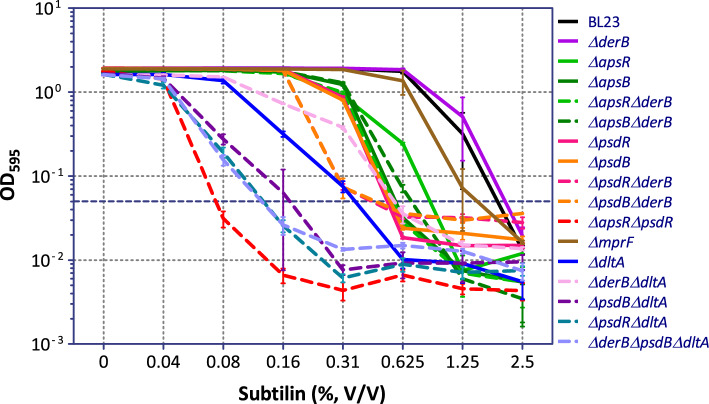
Effect of increasing concentrations of subtilin on the growth of *L. paracasei* BL23 and its derivative strains after 15 h of incubation. Strains were inoculated to an optical density at 595 nm (OD_595_) of 0.05 (horizontal dashed line) in MRS with different concentrations of subtilin (in %, volume/volume). Final OD_595_ readings were taken after 15 h of incubation at 37 °C (OD_595nm_ 15h). MIC_15H_ (see Table 1) was defined at the lowest antibiotic concentration where the final OD was at or below the starting OD. Means and standard deviations from six replica are presented.

The subtilin susceptibility of the single mutant strains agreed with our previous observations^17,18^ (Table 1). The absence of the regulatory components of either the Aps or the Psd systems increased the sensitivity of *L. paracasei* BL23 towards subtilin in a comparable level: *ΔpsdR, ΔpsdB, ΔapsR* and *ΔapsB* were around 2 to fourfold more sensitive than the parental strain (Table 1 and Fig. 1). As expected for Bce-like systems, where the ABC and the TCS form a functional unit, inactivation of either the TCS or the ABC transporter resulted in almost identical phenotypes, especially for the Psd system. Likewise, the impairment of the Dlt-system functionality increased the sensitivity of *L. paracasei* BL23 to subtilin since the *ΔdltA* strain was fourfold more sensitive than the parental strain. On the contrary, the contribution of MprF and DerAB to subtilin resistance was minor. While the absolute MIC values for subtilin (2.5%) were identical for *∆derB* and the wild type (Table 1), elimination of *derB* together with *∆psdB* (strain *ΔderBΔpsdB*) resulted in slightly reduced growth in the presence of the peptide relative to the corresponding parental strains (Fig. 1). This minor contribution of DerAB to subtilin resistance is in agreement with our previous results^18^. Finally, the *ΔmprF* strain had an MIC_15H_ value between 1.25 and 2.5%, showing a minor contribution to subtilin resistance as previously reported^17^.

**Table 1:** MIC_15H_ values of subtilin against *L. paracasei* BL23 and its derivative strains.

Strains	MIC_15H_ value^a^ (subtilin %, V/V)	Strains	MIC_15H_ value^a^ (subtilin %, V/V)
BL23	2.5	*ΔpsdR*	0.625
*ΔderB*	2.5	*ΔpsdB*	0.625
*ΔmprF*	1.25–2.5	*ΔderB ΔpsdR*	0.625
*ΔdltA*	0.625	*ΔderB ΔpsdB*	0.625
*ΔderB ΔdltA*	0.625	*ΔapsR*	1.25
*ΔpsdB ΔdltA*	0.16	*ΔapsB*	0.625
*ΔpsdR ΔdltA*	0.16	*ΔderB ΔapsR*	0.625
*ΔderB ΔpsdB ΔdltA*	0.16	*ΔderB ΔapsB*	1.25
*ΔpsdR ΔapsR*	0.08		

^a^MIC values were defined as the concentration of subtilin under which the growth of the strains was completely inhibited at the 15th hour of the cultivation.

Next, we tested the subtilin sensitivity of strains simultaneously deficient in the Psd and the Dlt system. Strains *ΔpsdBΔdltA* and *ΔpsdRΔdltA* were 15.6-fold more sensitive to subtilin than the wild type strain BL23, and fourfold more sensitive than the corresponding parental strains *ΔdltA* and *ΔpsdB* (Table 1). All together these results indicate that the PsdAB transporter and the Dlt system are the main subtilin resistance determinants in BL23. Finally, simultaneous inactivation of both RR rendered strain *ΔpsdRΔapsR* 31-fold more sensitive to subtilin than the wild type strain *L. paracasei* BL23. This result suggested that the subtilin response in *L. paracasei* BL23 is under dual control of the two paralogous systems, PsdRSAB and ApsRSAB, which together regulate all the subtilin resistance determinants. We next performed gene expression studies in response to subtilin to verify this hypothesis.

### Transcriptional response to subtilin: all the subtilin resistance determinants are solely under control of ApsR and PsdR

The transcriptional response of the genes encoding the subtilin resistance determinants was determined by qRTPCR after the exposure of exponentially growing cultures (OD_595_ ≈ 0.5) of BL23 and derived strains to a subinhibitory subtilin concentration of 0.02% (v/v), which had a significant inhibitory effect on exponentially growing cultures of the most sensitive strain *ΔpsdRΔapsR* (Table 1 and Supplementary Fig. S2), without completely inhibiting its growth.

**Figure 2:**
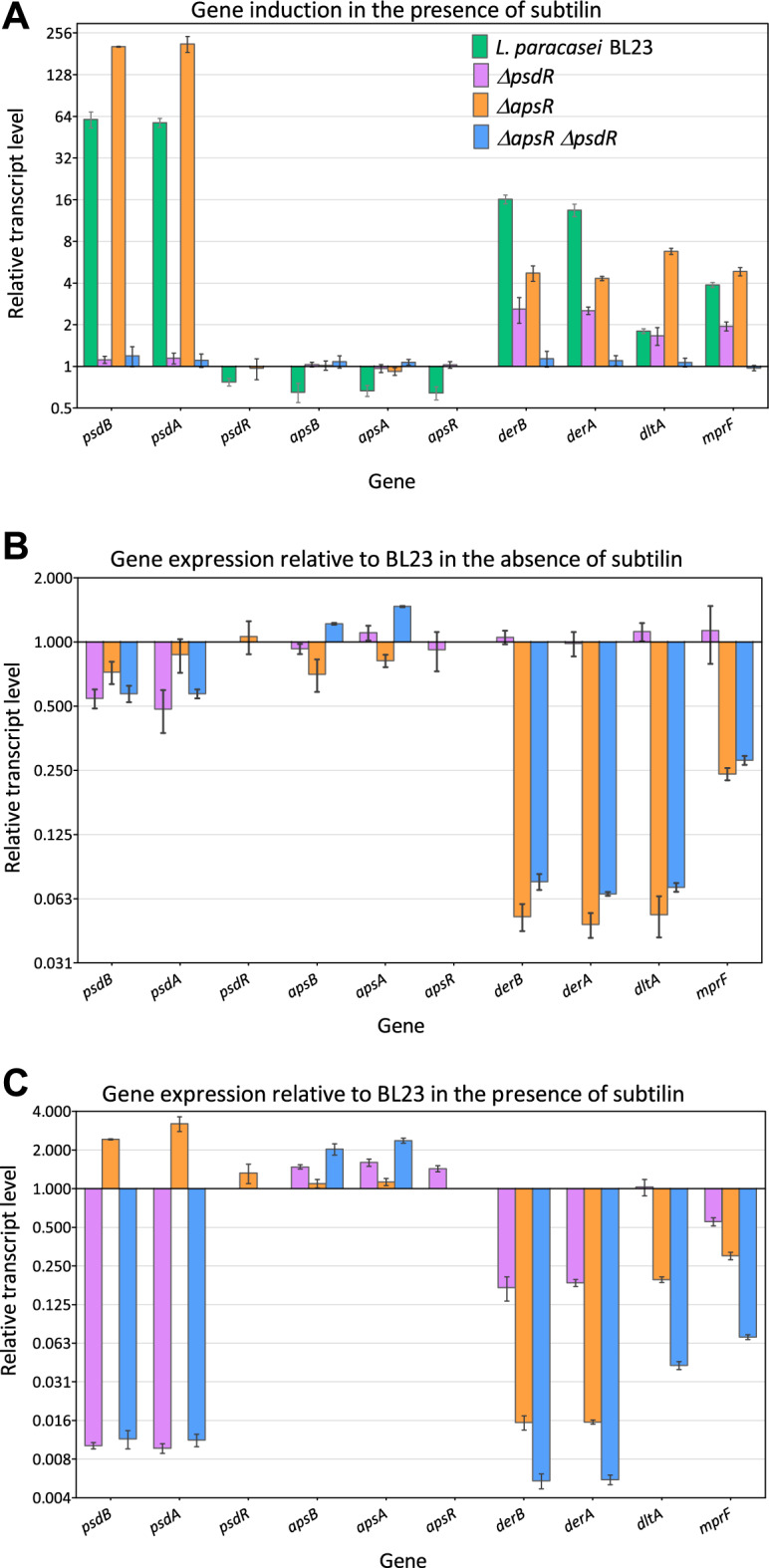
Gene induction in the presence of subtilin. The transcriptional response to subtilin of the *bce*-like genes, *dltA* and *mprF* in *L. paracasei* BL23 and derived strains was analyzed. Cell cultures at mid-exponential growth phase were treated with 0.02% (v/v) subtilin for 10 min. qRT-PCR was performed to determine the expression level of the genes indicated on the X-axis. (**A**) Relative transcript levels of the genes under study in *L. paracasei* BL23 and derivative strains 10 min after subtilin addition compared to the levels of the same strains in the absence of subtilin. Relative transcript levels of the same genes in *L. paracasei* BL23-derived strains are shown compared to the parental strain in the absence of subtilin (**B**) and 10 min after subtilin addition (**C**). The strains are color-coded as shown in graph (**A**). The means and standard deviations (error bars) were obtained from biological triplicates.

Subtilin exposure of BL23 strongly induced the expression of *psdAB* and *derAB* by around 60-fold and 16-fold, respectively. The expression of the *dlt* operon and the *mprF* were also activated, albeit to a lower degree (Fig. 2A). These induction patterns agree with our previous observations^18^. No induction of *psdR*, *apsR* and *apsAB* was observed, which is consistent with the role of PsdR, ApsR and ApsAB in mediating AMP sensing but not resistance against them^17^.

The *ΔapsR* strain showed a strong decrease of *derAB*, *dltA* and *mprF* expression in reference conditions (Fig. 2B), in agreement with our previous results^17^. Hence, ApsRS controls the expression of these three loci regardless of the presence of AMPs. While a strong decrease in the expression levels of these genes in *ΔapsR* compared to the parental strain was also observed after the addition of subtilin (Fig. 2C), their expression was still induced more than fourfold in the *ΔapsR* strain relative to reference conditions (Fig. 2A). A similar expression pattern was previously observed for *ΔapsR* in response to nisin^17^.

In the *ΔpsdR* mutant, only small differences in transcript levels relative to the wild type were observed in reference conditions (Fig. 2B). In contrast, inactivation of PsdR led to a complete loss of *psdAB* induction in response to subtilin (Fig. 2A and C). Interestingly, the expression of *derAB* and *mprF* was also reduced when compared with the expression levels in the wild type (Fig. 2A and C), suggesting that PsdR might affect the activity of the *derA* and *mprF* promoters.

Finally, we investigated the transcriptional response of the double mutant *ΔpsdRΔapsR* to subtilin. In reference conditions, the decrease in the expression of *derAB*, *dltA* and *mprF* in the *ΔpsdRΔapsR strain* relative to the wild type was similar to that observed for the *ΔapsR* strain (Fig. 2B). This can be explained by the absence of the primary regulator ApsR. In response to subtilin, inactivation of both genes resulted in the complete loss of induction of all genes regulated by either RR, namely, *psdA, psdB, derA, derB, dltA* and *mprF* genes (Fig. 2A). The loss of induction of the *psdA* and *psdB* genes was similar in the *ΔpsdRΔapsR* and the *ΔpsdR* strains (Fig. 2A and C), supporting an exclusive regulation of the expression of *psdAB* by PsdR. However, the expression rates in the *ΔpsdRΔapsR* strain were threefold lower for *derA* and *derB,* and around 4.5-fold lower for *dltA* and *mprF,* than in the *ΔapsR* strain (Fig. 2C), indicating an additive effect of both RRs on the expression of Aps-dependent genes.

### Aps-genes are also cross-regulated by PsdR in response to nisin

Previous results had shown that mutants in the ApsRSAB system had a much lower level of expression of the *dlt* operon and the *mprF* gene compared to that of the wild type. But a minor nisin-dependent induction of both systems relative to reference conditions was still observed^17^, indicating that additional regulatory systems might control the expression of the *dlt* operon and *mprF* in response to nisin in the absence of a functional ApsRSAB module.

**Figure 3:**
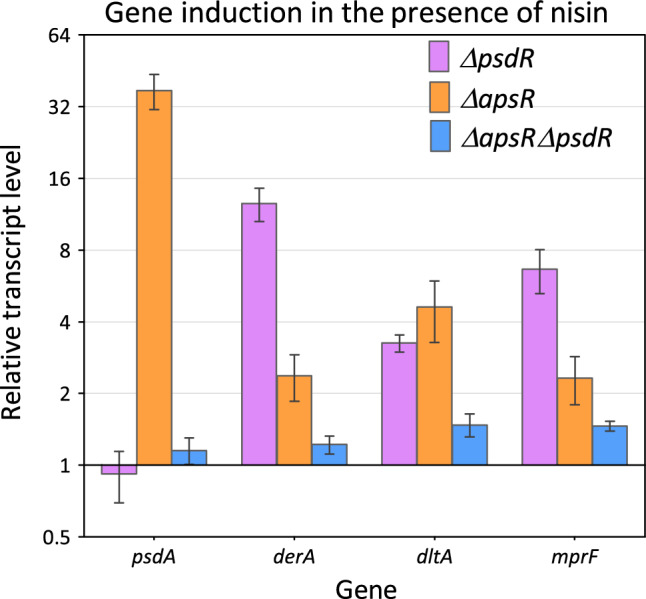
Gene induction in the presence of nisin. The transcriptional response to nisin of the *bce*-like resistance genes, *dltA* and *mprF* in *ΔpsdRΔapsR* was analyzed*.* Cell cultures at mid-exponential growth phase were treated with 22.5 ng ml^−1^ nisin for 10 min and qRT-PCR was performed to determine the expression level of the genes indicated on the X-axis. Relative transcript levels of the genes 10 min after nisin addition were compared to the levels of the same strain in the absence of nisin. The means and standard deviations (error bars) were obtained from biological triplicates. For comparison, the gene expression profiles in response to the same concentration of nisin of the strains *ΔpsdR* and *ΔapsR* previously reported^17^ are also presented.

We therefore decided to investigate if PsdR also cross-regulates ApsR-target genes in response to nisin. The MIC_15H_ for nisin were determined as 0.5 µg/ml for BL23, 0.3 µg/ml for *ΔpsdR* , 0.2 µg/ml for *ΔapsR* and 0.1 µg/ml for *ΔpsdRΔapsR.* Simultaneous inactivation of both RRs in the *ΔpsdRΔapsR* mutant rendered *L. paracasei* 2–threefold more sensitive to nisin than the single *ΔapsR* and *ΔpsdR* mutant strains, and fivefold more sensitive to nisin than the wild type. Quantification of the gene expression of the *ΔpsdRΔapsR* mutant in response to nisin showed that inactivation of both genes resulted in the complete loss of induction of the genes regulated by either RR, namely, *psdA, derA, dltA* and *mprF* genes (Fig. 3).

Taken together, these results indicate that the expression of all subtilin and nisin resistance determinants investigated is exclusively under the transcriptional control of the two paralogous Bce-like systems PsdRS and ApsRS. Moreover, our results suggest that cross-regulation in response to subtilin and nisin might occur between the RR PsdR and the ApsR-regulated promoters in the absence of ApsRSAB, but not vice versa. This second idea implies that PsdR can bind to the ApsR-regulated promoters. We therefore investigated this possibility in more detail, initially by comparing the predicted quaternary structures of PsdR and ApsR.

### System comparison: the predicted structures of the paralogous response regulators ApsR and PsdR are very similar

**Figure 4:**
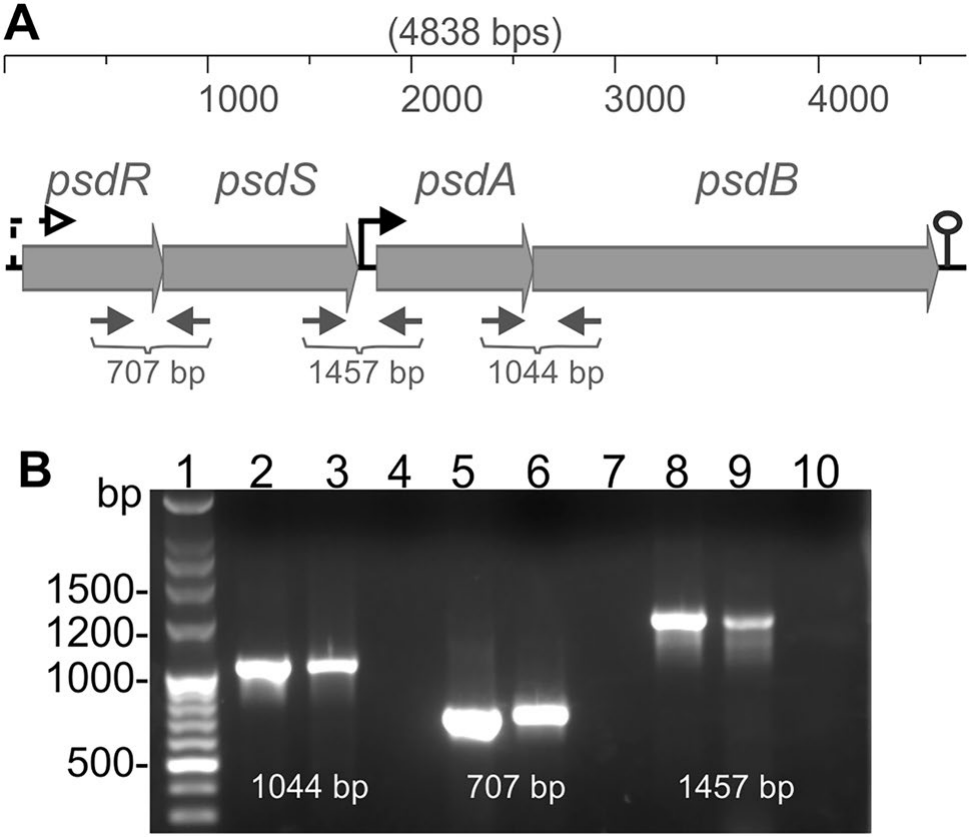
Schematics of the *psdRSAB* operon of *L. paracasei* BL23 (**A**) and RT-PCR analysis of transcripts (**B**). (**A**) Genes are drawn as grey filled arrows; the thin grey arrows below the genes indicate the position and the product size of the cross-gene primers used for the RT-PCR presented in (**B**); the position of a putative terminator is indicated by a lollipop; the Bce-like promoter is indicated by a solid black bent arrow and the putative constitutive promoter driving the expression of the full operon is indicated as a dashed black bent arrow. (**B**) The PCR amplification products correspond to the following cross-gene primers: RG082–RG083 (lines 2–4, amplification of intergenic region *psdB-psdA*, product size [1044 bp]); RG078–RG079 (lines 5–7, amplification of intergenic region *psdS-psdR* [707 bp]); and RG080–RG081 (lines 8–10; amplification of intergenic region *psdA-psdS* [1457 bp]); line 1 contains the molecular weight ladder. Negative controls using RNA as template are presented in lines 4, 7 and 10 to verify the absence of residual genomic DNA in the purified RNA samples. Positive controls amplified from genomic DNA are presented in lines 2, 5 and 8. Lines 3, 6 and 9 contain the RT-PCR products amplified from the cDNA. A cropped gel is depicted for clarity; the original gel picture can be found in Supplementary Fig. S4.

While the ApsRSAB module contains a sensing transporter not directly involved in AMP detoxification, the PsdRSAB module is an archetypical Bce-like stand-alone detoxification system, in which the transporter is involved both in *sensing of* and mediating *resistance against* the AMPs. Both systems are also dissimilar in their genetic organization: *apsRS* and *apsAB* are coded in opposite directions, while the *psdRSAB* genes are organized in an apparent four-gene operon with a consensus promoter binding sequence for Bce-like response regulators^9^ located upstream of the *psdAB* genes^17^. RT-PCR with cross-gene primers designed to amplify the intergenic regions between *psdS-psdR*, *psdA-psdS* and *psdB-psdA* confirmed that the four genes can be cotranscripted in a single polycistronic mRNA (Fig. 4). This expression of the complete *psdRSAB* operon must be driven by an additional, and so far unidentified, promoter sequence upstream of *psdR*.

**Figure 5:**
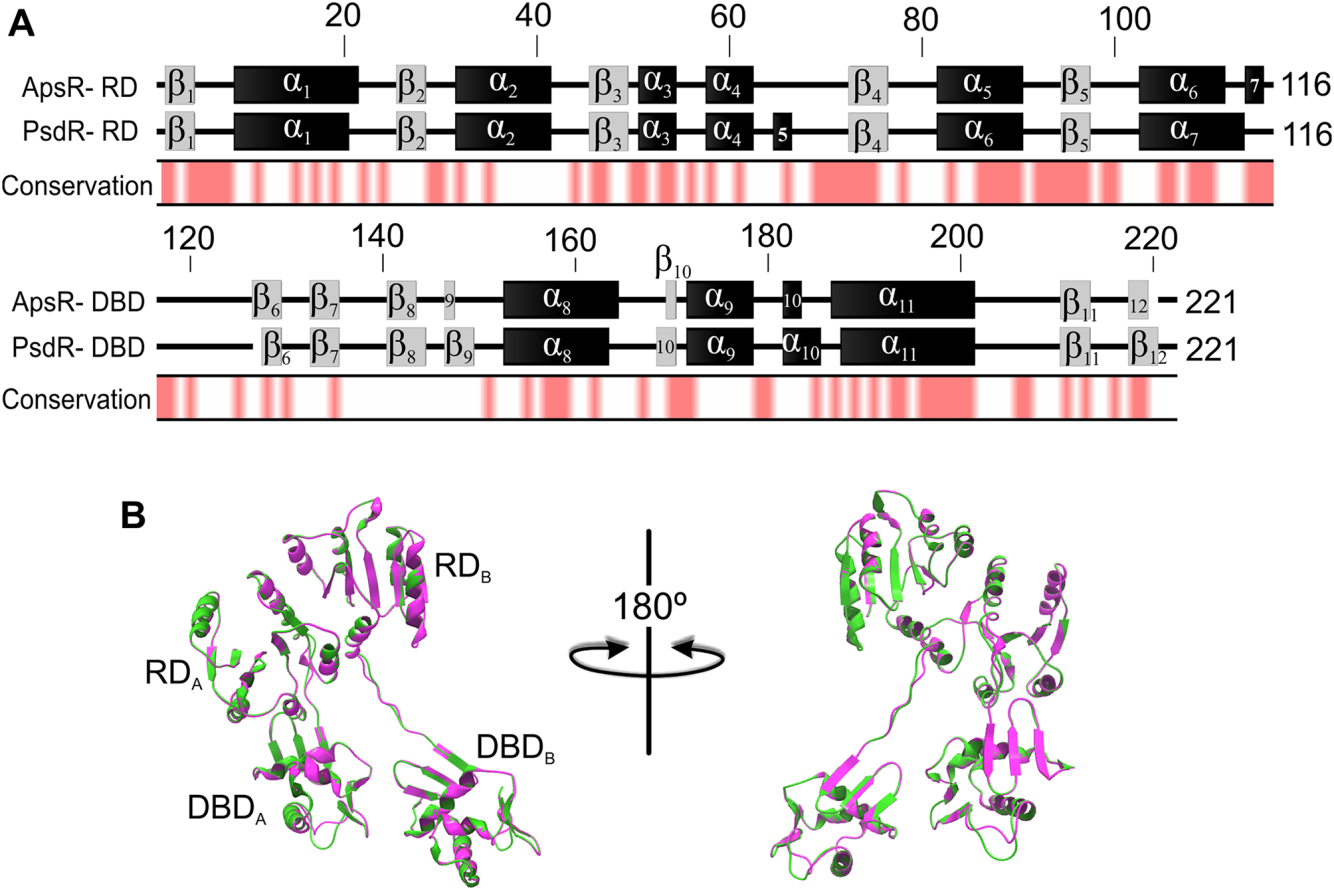
Comparison of the primary and the predicted secondary and quaternary structures of the paralogous response regulators ApsR and PsdR from *L. paracasei* BL23. (**A**) The secondary structure of ApsR and PsdR receiver domains (RD) and DNA binding domains (DBD) based on predictions by Swiss Model^30^ is displayed as α-helices (black boxes) and β-sheets (grey boxes). Amino acids positions are indicated on top of the sequences. Conserved amino acids between both response regulators primary sequences are indicated in red under the secondary structures. (**B**) Ribbon representation of overlaying quaternary structures of ApsR (pink) and PsdR (green) based on predictions by Swiss Model^30^ showing the conservation of their predicted structural organizations. The RRs are displayed as asymmetric homodimers in an active-like conformation (see Materials and methods for further detail on the crystal structures used as templates for homology modeling). The positions of the receiver domains (RD) and the DNA binding domains (DBD) from each monomer are indicated.

Regarding the two paralogous response regulators PsdR and ApsR from *L. paracasei* BL23, they belong to the OmpR/PhoB subfamily, and their protein sequences are 42% identical (Fig. 5). Based on predictions through the Swiss Model web portal^30^, their receiver domains are predicted to contain a central parallel β-sheet surrounded by α-helices—a topology typical for receiver domains^31^—and the C-terminal DNA binding domains are predicted to contain three/four α-helices and two antiparallel β-sheets (Fig. 5). Overall, the predictions indicate that PsdR and ApsR might adopt very similar quaternary structures, which only differ partly in the length of some secondary motifs (Fig. 5). We previously reported the high similarity between the ApsR-regulated promoters (P_*derA*_, P_*mprF*_ and P_*dltA*_) and the PsdR-regulated promoter (P_*psdA*_), all sharing a consensus sequence AnnTTACnAnnnnGTnAG^17^. Hence, both the predicted quaternary structures of PsdR and ApsR, and the predicted promoter sequences recognized by them are very similar. We therefore next addressed the question of whether cross-regulation of ApsR-dependent promoters by PsdR, and vice versa, of the PsdR-regulated promoter by ApsR, could be possible. Towards this goal, we performed RR-DNA in vitro binding studies.

### PsdR binds in vitro with similar affinities to all the Bce-like, including all the ApsR-regulated, promoters

Biolayer interferometry allows the identification of molecular interactions in real time by optical sensing. It was used to characterize ApsR- and PsdR-binding to their target promoters. No binding was detected with unphosphorylated proteins (not shown). ApsR could be phosphorylated with acetyl-phosphate, whereas no phosphorylation of PsdR was detected. When ammonium phosphoramidate was used, both RR could be phosphorylated. However, no binding could be detected with ApsR phosphorylated with ammonium phosphoramidate. Therefore, results reported below were obtained with ApsR phosphorylated with acetyl-phosphate and PsdR phosphorylated with ammonium phosphoramidate.

ApsR-P bound the four promoters, whereas no binding was detected to the Flta negative control DNA fragment (Table 2). Apparent affinity constants showed a preferential binding of ApsR-P in the order P_*derA*_ > P_*dlt*_ > P_*mprF*_ > P_*psdA*_ under our experimental conditions. This preference for binding broadly agrees with the genetic evidence obtained by RT-qPCR in previous studies^17,18^ and in this study. It is worth noting that expression of DerAB consistently showed a stronger induction than Dlt or MprF in response to either subtilin (Fig. 2) or nisin^17,18^. PsdR-P also bound the four cognate promoters, while again no binding to the negative control DNA fragment was detected (Table 2). In contrast to ApsR, similar apparent affinity constants were measured for the four promoters under our experimental conditions. This result is at odds with the evidence obtained by RT-qPCR, where a clear preference of PsdR for the P_*psdA*_ promoter could be inferred.

**Table 2:** Biolayer interferometry kinetics values of ApsR and PsdR interaction with DNA fragments encompassing predicted Bce-like binding sites and a negative control.

Protein	DNA fragment	K_D_ (*k*_d_/k_a_) (M)^1^	*k*_a_ (M^−1^ s^−1^)^1^	*k*_d_ (s^−1^)^1^
ApsR^2^	P_*psdA*_	1.084 × 10^−4^	1.213 × 10^3^ ± 5.997 × 10^2^	1.314 × 10^−1^ ± 4.232 × 10^−3^
	P_*derA*_	4.743 × 10^−6^	7.962 × 10^3^ ± 3.669 × 10^2^	3.777 × 10^−2^ ± 1.133 × 10^−3^
	P_*dlt*_	7.470 × 10^−6^	2.611 × 10^3^ ± 9.089 × 10^1^	1.951 × 10^−2^ ± 3.097 × 10^−4^
	P_*mprF*_	2.664 × 10^−5^	1.888 × 10^3^ ± 1.857 × 10^2^	4.993 × 10^−2^ ± 6.945 × 10^−4^
	Flta	ND^3^	ND	ND
PsdR^4^	P_*psdA*_	1.156 × 10^−7^	2.539 × 10^4^ ± 5.066 × 10^2^	2.934 × 10^−3^ ± 8.383 × 10^−5^
	P_*derA*_	1.720 × 10^−7^	2.372 × 10^4^ ± 4.221 × 10^2^	4.078 × 10^−3^ ± 8.554 × 10^−5^
	P_*dlt*_	1.002 × 10^−7^	2.612 × 10^4^ ± 3.371 × 10^2^	2.617 × 10^−3^ ± 4.249 × 10^−5^
	P_*mprF*_	9.482 × 10^−8^	3.186 × 10^4^ ± 3.397 × 10^2^	3.021 × 10^−3^ ± 4.018 × 10^−5^
	Flta	ND^3^	ND	ND

^1^K_D_, affinity constant, k_a_, association rate constant, k_d_, dissociation rate constant. ^2^ApsR phosphorylated with acetyl-phosphate. The binding assays with ApsR phosphorylated with phosphoramidate failed for unknown reasons. ^3^Not detected. The protein did not bind the DNA fragment under our experimental conditions. ^4^PsdR phosphorylated with ammonium phosphoramidate. PsdR could not be phosphorylated with acetyl-phosphate for unknown reasons.

## Discussion

In previous reports, we described the response of the two Bce-like systems of *L. paracasei* BL23, PsdRSAB and ApsRSAB, to different AMPs^15,17,18^. In this work, we specifically analyzed their response to the lantibiotic subtilin. We tested the subtilin sensitivity of a collection of single, double and triple mutants in the BceAB-like ABC transporters DerAB, PsdAB and ApsAB; the BceRS-like TCS PsdRS and ApsRS; and the AMP-resistance determinants Dlt system and the MprF protein. We also quantified the transcriptional response to subtilin of the genes encoding these systems in strains lacking one (*ΔpsdR* and *ΔapsR* strains) or both (*ΔpsdRΔapsR*) RR. The sensitivity assays showed that the ABC transporter PsdAB together with the Dlt system are the primary subtilin resistance determinants (Table 1 and Fig. 1). The transcriptional studies indicate that this subtilin response is regulated uniquely by the two paralogous systems PsdRSAB and ApsRSAB (Fig. 2). Remarkably, (i) the expression of *derA*, *derB* and *mprF* genes decreased in the *ΔpsdR* relative to the parental strain in response to subtilin (Fig. 2C), (ii) the expression of the same three genes plus *dltA* decreased in the *ΔpsdRΔapsR* strain when compared with the relative expression in the *ΔapsR* also in response to subtilin (Fig. 2C), (iii) a minor but significant induction of these genes in response to subtilin was observed in the *ΔapsR* strain but was lost in the *ΔpsdRΔapsR* strain relative to reference conditions (Fig. 2A), and (iv) similar results were obtained in response to nisin (Fig. 3 and^17^).

Taken together, these results point towards a PsdR-dependent cross-regulation of *derAB*, *mprF* and *dlt-*operon genes in response to subtilin and nisin. The high similarity of the predicted structures of both RR PsdR and ApsR (Fig. 5), and their cognate DNA binding sites within the P_*psdA*_, P_*derA*_, P_*mprF*_ and P_*dltA*_ promoter sequences^17^ argued in favor of this possibility. We further investigated it by performing RR-DNA binding studies. The biolayer interferometry results show a preferential binding of ApsR-P in the order P_*derA*_ > P_*dlt*_ > P_*mprF*_ > P_*psdA*_*,* which is in agreement with previous gene expression studies, using the *ΔapsR* strain in response to nisin and subtilin. In contrast, PsdR-P bound with similar apparent affinity constants to all four promoters, although gene expression analyses indicated a preferential binding for P_*psdA*_ in vivo (Table 2). Furthermore, K_D_ values determined for PsdR where at least one order of magnitude lower than those determined for ApsR. Several reasons may account for these discrepancies. First, the amount of purified proteins actually active could not be determined for these assays. In addition, the same phosphorylating agent could not be used for both RRs as PsdR was not phosphorylated by acetyl-phosphate and no binding activity was observed with ApsR phosphorylated with ammonium phosphoramidate. Besides, we observed that the unphosphorylated proteins were not active but, phosphorylation with either acetyl-phosphate or ammonium phosphoramidate was not complete and use of the latter phosphorylating agent resulted in additional phosphorylations as evidenced by the detection of additional bands in SDS-acrylamide gel electrophoresis (see Supplementary Fig. S3). Additional phosphorylations of RRs by small phosphor-donors such as acetyl-phosphate and phosphoramidate had been previously observed although their effects on the binding activity have not been determined^32^. All these factors make comparative analysis between the results obtained for each RR in the bilayer interferometry assay difficult. Notwithstanding, the results obtained with ApsR phosphorylated with acetyl-phosphate agree with the genetic evidence obtained, thus supporting that ApsR observed preference for the different promoters possibly reflects the situation in vivo.

Within the Bce-like systems, some in vivo cross-talk has been described in *B. subtilis* by cross-phosphorylation of the PsdR RR by the BceS HK^33^. Whereas cross-talk is defined as “detrimental communication between two different signaling pathways”, cross-regulation has been previously described as “communication between distinct signaling pathways that provides a physiological benefit to the organism”^2^. Altogether, our new results indicate that the Bce-like systems from *L. paracasei* BL23 possess cross-regulation at the level of RR-promoter interaction, which allows the existence of a hidden potential in the response of these Bce-like systems to certain AMPs, as discussed next.

**Figure 6:**
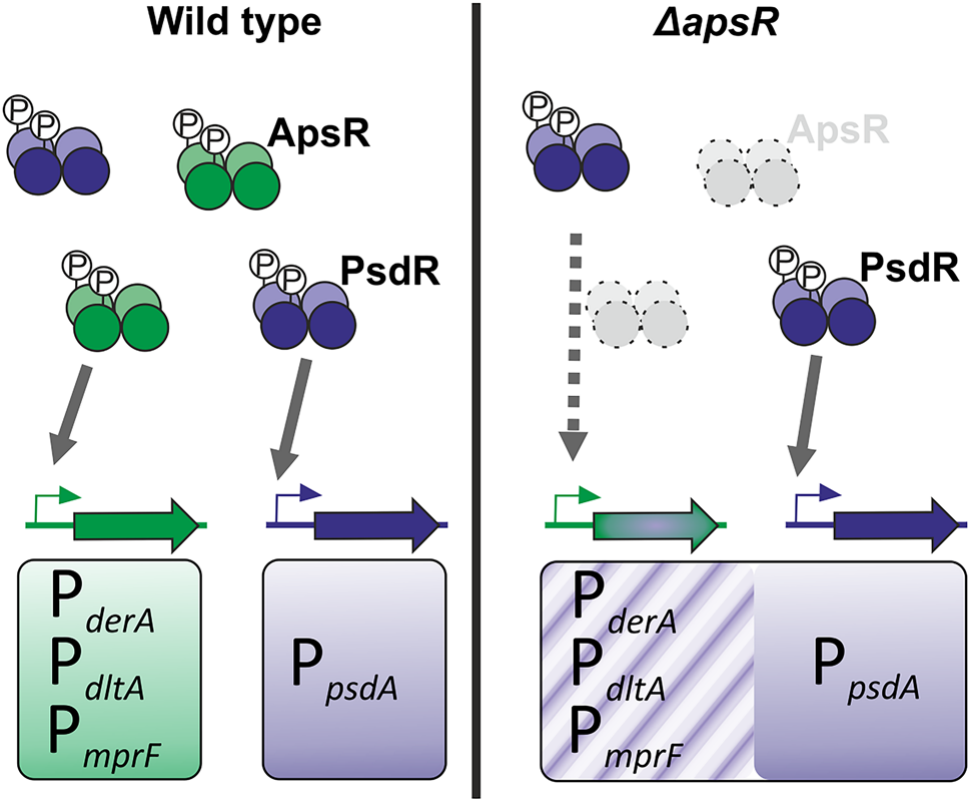
Model for the cross-regulation at the RR-promoter interaction within *L. paracasei* BL23 Bce-like systems. ApsR and PsdR transcriptional regulation of their target promoters are indicated in green and blue, respectively. Homodimers of phosphorylated-RRs are indicated; target promoters are depicted as grey and blue bent arrows, and regulated genes as green and blue filled arrows. ApsR and PsdR specifically recognize their target promoters in the wild type strain (panel on the left, grey solid arrows). A genetic perturbation, such as the elimination of the ApsR response regulator in the *∆apsR* strain, unveils the cross-regulation of the *apsR*-promoters by the PsdR response regulator (panel on the right, grey dotted arrow).

Natural regulatory redundancy in the way of paralogous/homologous RRs controlling overlapping regulons in response to different stimulus is rare but can be of biological relevance. It might be used for fine-tuning the expression of target genes as a function of the “amount” and/or “type” of stimulus. Examples of this situation are described in other species (reviewed in^4^). In *Escherichia coli*, the expression of several operons in response to nitrate and nitrite is regulated by the homologous response regulators NarL and NarP. Both RRs recognize the same consensus heptameric DNA-binding sequence. However, some operons are regulated by both RRs, while others only respond to NarL—depending on the base-pair spacing between the heptameric inverted repeats^34^. In *Desulfovibrio vulgaris,* studies mapping the gene targets of its RRs showed that the paralogous RRs DVU0539 and DVU0946 share the same binding motifs on their target promoters but regulate the expression of their target genes differentially^35^. In *Pseudomonas aeruginosa*, the OmpR/PhoB subfamily regulators PhoB and TctD inversely regulate the expression of a common subset of genes as a function of the phosphate availability and the carbon source^36^. In *L. paracasei*, however, the cross-regulation of Aps-promoters by PsdR was unveiled only by the gene expression studies in the event of a genetic perturbation (*∆apsR*) (Fig. 6). The sensitivity phenotypes to different AMPs of the *∆apsR* strain show that PsdR alone is not able to physiologically compensate for the absence of ApsR. Even though the results presented herein do not allow to unequivocally determine if this cross-regulation also happens in a natural cellular setting with both RRs present in the cell, it is tempting to speculate that this cross-regulation might be a way of boosting the expression of the *dlt*-operon, involved in maintenance of critical cell membrane functions^19^, and DerAB, a more specific detoxification system^18^.

We previously reported that nonproductive signaling interference due to cross-talk between DerAB transporter and PsdRS TCS results in a diminished response of the PsdRSAB system to nisin in vivo. Consequently, the *derB* deletion mutant is hyperresistant against nisin. This is mostly due to the increased activation of *psdAB* expression once the signaling interference is removed and the full signaling potential within the Psd system is released^18^. The newly described cross-regulation adds to this hidden potential in the response of these Bce-like systems to certain AMPs, as an additional resistance phenotype orchestrated by the Bce-like systems from BL23. Interestingly, other studies with Bce-like systems from other bacteria also unmasked the intrinsic potential of these AMP detoxification systems upon genetic perturbations. In *Staphylococcus aureus*, NsaS is the sensor HK of the TCS NsaRS, which controls the expression of VraDE, an ABC transporter involved in nisin detoxification. Gain-of-function mutants in NsaS overexpress VraDE; the full potential activation of this nisin-detoxification system results in an acquired nisin resistance phenotype^37,38^. Of note, in all cases the full response of a “silent” intrinsic detoxification system is only unmasked once a genetic perturbation in a regulatory system is artificially introduced. It could be possible that these mechanisms remain naturally silent to prevent an over-reaction in the response to these AMPs, which could result in a high fitness cost for the cells. AMPs are considered as new alternatives to classical antibiotics due to lower levels of natural resistance development being reported^39,40^. While this is not the case for *L. paracasei*, the results with the Bce-like systems from pathogenic species such as *S. aureus* suggests that we need to remain vigilant for the development of antimicrobial resistances upon the prolonged clinical use of AMPs. Gain-of-function mutations in regulatory circuits could be selected upon repeated exposition to the antimicrobials, as have been described in daptomycin-resistant *Enterococcus*^41–43^. Future investigations should focus on the search for molecular inhibitors of such signaling pathways, as well as on chemically modified AMP-derivatives not recognizable by the corresponding bacterial detoxification systems.

In summary, this report adds to the detailed understanding of Bce-like systems from *L. paracasei*. Our results indicate that the Psd system can cross-regulate Aps-target genes. This observation uncovers additional features of these signaling pathways and will help to better understand complex regulatory circuits involved in mediating bacterial antibiotic resistance. Our results may help studying similar systems in pathogenic Firmicutes.

## Materials and methods

### Bacterial strains and growth conditions

The strains used in this study are listed in Supplementary Table S1. *Lacticaseibacillus paracasei* BL23 (formerly *Lactobacillus casei* BL23^44^) and the derivative mutant strains were routinely grown in MRS (deMan, Rogosa and Sharpe) broth (Difco™ Lactobacilli MRS Broth: proteose peptone no.3 10 g l^−1^, beef extract 10 g l^−1^, yeast extract 5 g l^−1^, dextrose 20 g l^−1^, polysorbate 80 1 g l^−1^, ammonium citrate 2 g l^−1^, sodium acetate 5 g l^−1^, magnesium sulfate 0.1 g l^−1^, manganese sulfate 0.05 g l^−1^, dipotassium phosphate 2 g l^−1^; Becton, Dickinson and Company, Sparks, USA) at 37 °C under static conditions. Erythromycin (Sigma-Aldrich, Merck KGaA, Darmstadt, Germany) at a final concentration of 5 µg ml^−1^ was supplemented into the media when required. *Escherichia coli* DH10β was used as an intermediate host for cloning purposes. *E. coli* strains were grown in LB-Broth (Luria/Miller) for molecular biology (tryptone 10 g l^−1^, yeast extract 5 g l^−1^, NaCl 10 g l^−1^, and pH 7.0; Carl Roth, Karlsruhe, Germany) at 37 °C with aeration, supplemented with ampicillin (Carl Roth, Karlsruhe, Germany) 100 µg ml^−1^ if required. The subtilin-producing strain *Bacillus subtilis* ATCC 6633 was kept on LB-plates and it was grown aerobically in the high sucrose-containing liquid media, Medium A (sucrose 100 g l^−1^, citric acid 11.7 g l^−1^, Na_2_SO_4_ 4 g l^−1^, (NH_4_)_2_HPO_4_ 4.2 g l^−1^, yeast extract 5 g l^−1^, salt mixture [KCl 7.62 g l^−1^, MgCl_2_·6H_2_O 4.18 g l^−1^, MnCl_2_·4H_2_O 0.543 g l^−1^, FeCl_3_·6H_2_O 0.49 g l^−1^, ZnCl_2_ 0.208 g l^−1^] 100 ml l^−1^, and pH adjusted to 6.8–6.9 with NH_4_OH (Ammonia solution)) to harvest subtilin-containing supernatant^45^. For plating, the media were supplemented with 1.5% (w/v) agar (Agar–Agar Kobe I, Carl Roth, Karlsruhe, Germany).

### Construction of *L. paracasei* mutant strains

Two methods were applied to mutate genes in *L. paracasei* BL23: (1) clean mutations leading to markerless removal of the complete target genes, (2) insertional mutations interrupting the target genes by the insertion of an erythromycin resistance cassette. Cloning in *E. coli* was performed following standard methods^46^. Chemically competent *E. coli* cells were transformed by the heat shock method. The vectors and plasmids used in this study are listed in Supplementary Table S1. Oligonucleotides used in this study are listed in Supplementary Table S2.

To generate markerless clean mutants, the up- and down-flanking fragments of the target gene were first amplified by PCR, and then fused into one piece by PCR. The fusion product was digested with restriction enzymes and ligated into the integrative vector pRV300 digested with the same enzymes by following the manufacturers’ instructions. The resulting construct was verified by PCR and DNA sequencing, and subsequently introduced into *L. paracasei* BL23 or derivative strains by electroporation^47^. Single-crossover integration was first selected by resistance to erythromycin; the insertion into target gene was confirmed by PCR. Secondly, one single-crossover integrant was grown in MRS media without erythromycin to allow a second crossover recombination leading to the elimination of the target gene. Cells were plated on MRS and replica plated on MRS plus erythromycin. Antibiotic-sensitive clones were isolated, and the loss of the target region was verified by PCR and DNA sequencing.

To generate the insertional mutants, an internal region of the target gene was amplified by PCR and ligated into vector pRV300 following the same procedure as described above. The resulting construct was then transformed into *L. paracasei* recipient strains. Desired mutants in which the plasmid was inserted via single-crossover into the target region were selected by resistance to erythromycin. The interruption of the target gene was verified by PCR.

### Subtilin production

Collection of subtilin-containing supernatant was performed as previously described^45^. Briefly, a single fresh colony of *B. subtilis* ATCC 6633 strain was inoculated into 25 ml of Medium A, and it was grown at 37 °C with agitation to promote subtilin production. The culture was collected after 24 h of growth, and it was centrifuged at 10,000×*g* for 15 min. The subtilin containing supernatant was filter-sterilized using 0.45 µm membrane filter (Sarstedt, Germany) to remove the remaining bacterial cells. The supernatant was aliquoted, heated up at 80 °C for 20 min and stored at −20 °C until use. Culture supernatant was considered to be at a concentration of 100% (vol/vol) for MIC assays.

### Sensitivity assays of *L. paracasei* BL23 and mutants towards subtilin and nisin

The susceptibility of *L. paracasei* BL23 and its variants to subtilin was examined by treating the strains with a series of dilutions of the same stock of subtilin-containing supernatant. Nisin from *Lactococcus lactis* (Sigma-Aldrich) was used for the nisin sensitivity assays. The procedure was performed following previously described protocols^17^. Briefly, overnight cultures of the strains under study were prepared with antibiotic selection when required, but no antibiotic was added for the Minimal Inhibitory Concentration (MIC) assays. Cells were harvested by centrifugation and washed twice using two volumes of 0.1% (w/v) peptone-water. After the last washing, the cell pellet was resuspended in MRS to a final OD_595_ ≈ 0.1. The cells were dispensed in 96 well plates to a final OD_595_ of 0.05, and serial-dilutions of subtilin or nisin in MRS were added to reach the desired final concentrations. MRS media was added to the reference cultures. Growth at 37 °C without shaking was monitored for 24 h using a Synergy™ NEO multi-mode microplate reader from BioTek® (Winooski, VT, USA). The MIC_15H_ was defined as the lowest concentration of antibiotic that completely inhibited bacterial growth (the final OD was at or below the starting OD) at 15 h^18^. The experiments were performed at least in triplicate.

### Sample collection for RNA isolation.

The subtilin concentration used for the transcriptomic studies was determined by exposing mid-exponential growing cultures (OD_595_ ≈ 0.5) to a series of subtilin dilutions, with one untreated control sample. The effect on growth was determined by measuring the OD_595_ every 45 min for more than 3 h. Selection of the subinhibitory concentration of subtilin was based on the phenotype of the most sensitive mutant *ΔpsdRΔapsR*, so that addition of subtilin had a significant effect on the growth rate of the culture without completely inhibiting it (Supplementary Fig. S2).

For RNA extraction, cultures at OD_595_ 0.5 were split in two halves: one was treated with the selected concentration of subtilin while the other one remained untreated as control sample. Incubation was continued for 10 min at 37 °C and the induction was stopped immediately by placing the tubes in an ice:NaCl (3:1, v/v) cooling bath. The cells were then harvested by centrifugation for 10 min at 5000×*g*, 4 °C. The cell pellets were snap-frozen in liquid nitrogen and stored at −80 °C until RNA isolation.

For nisin, sample collection was performed as previously described and a concentration of 22.5 ng ml^−1^ was used for comparative purposes^17^.

Three replicas of each condition were harvested.

### Cell lysis and RNA isolation.

Frozen cell pellets were resuspended in 200 µl of killing buffer (20 mM Tris/HCl pH 7.5; 5 mM MgCl_2_; 20 mM NaN_3_) and they were subsequently disrupted for 2 min at 2600 rpm in a liquid nitrogen pre-cooled homogenizer (Mikro-Dismembrator S, Sartorius, Germany). Next, the cell powder was resuspended in lysis buffer pre-warmed at 50 °C (4M guanidine-thiocyanate; 0.025 M NaOAc, pH5.2; 0.5% N-lauroylsarcosinate; and DEPC treated H_2_O) and transferred into 2 ml reaction tubes. One volume of Phenol Mix (Phenol : Chloroform : Isoamylalcohol, 25 : 24 : 1, pH 4.5–5 (ROTI®Aqua-P/C/I, for RNA extraction, Carl Roth, Germany)) was added and the mixtures were vigorously shaken for 5 min. The samples were centrifuged for 5 min at 12,000×*g* and supernatants were transferred to new tubes. The treatment with phenol mix was performed twice and the same procedure was performed with 1 volume of chloroform mix (Chloroform : Isoamylalcohol, 24 : 1 (Roti®-C/I, for nuclear acid extraction, Carl Roth, Germany)). Next, 1/10 volume of NaOAc (3 M, pH 5.2) and one volume of isopropanol were added to the supernatants. The samples were mixed by inverting and they were incubated at −80 °C overnight to allow RNA precipitation. On the next day, the samples were centrifuged (30 min, 15.000×*g*, 4 °C), supernatants were discarded, the pellets were washed twice with 1 ml of 70% ethanol, and collected by centrifugation (5 min, 15.000×*g*). Supernatants were removed carefully, and the pellets containing the nucleic acids were dried for 10 min at room temperature, prior to their resuspension in 50 µl of DEPC treated H_2_O. Nucleic acids were stored at −80 °C until use.

### Reverse transcription and quantitative real-time PCR (qRT-PCR)

The purity and yield of the extracted RNA were determined using Nanodrop® 1000 Spectrophotometer (Thermo Fisher Scientific); the integrity was further checked by agarose gel electrophoresis. Genomic DNA was removed using Ambion™ DNase I (RNase-free) (Invitrogen™, Thermo Fisher Scientific) following manufacturer’s instructions. Synthesis of cDNA was performed using SuperScript® VILO™ cDNA Synthesis Kit (Invitrogen™, Thermo Fisher Scientific) following the instructions of the manufacturer. qRT-PCR were carried out with Luna® Universal qPCR Master Mix (New England BioLabs) using a REAL-TIME PCR thermocycler qTOWER3 (Analytik Jena AG, Jena, Germany); the instrument was controlled by the program of qPCRsoft version 3.4 (Analytik Jena AG, Jena, Germany). The differential gene expression was quantified using the 2–∆∆CT (Livak) Method. All the primers used are listed in Supplementary Table S2. *lepA*, *ileS*, *pyrG*, and *pcrA* were used as constitutive reference genes^48^. Linearity and amplification efficiency for each primer pair were previously determined^17^.

*Detection of polycistronic transcripts of the* psdRSAB* operon.*

Total RNA samples from exponentially growing cultures (OD_595_ of 0.5) of *L. casei* BL23 exposed for 10 min to a sublethal concentration of nisin (22.5 ng ml^−1^) were isolated following previously described protocols ^17,48^. The RNA samples were treated with the Ambion Turbo DNA-free kit (Applied Biosystems) using the routine DNase I treatment outlined by the supplier to remove genomic DNA. The Experion automated electrophoresis system (Bio-Rad) was used to evaluate the quality and concentration of the RNA samples and those with 23S/16S ratios lower than 0.85 were discarded. First-strand cDNA was synthesized from 1 µg RNA using the SuperScript VILO cDNA synthesis kit (Invitrogen) as recommended by the manufacturer. Inter-gene PCRs were performed to amplify the cDNA, genomic DNA (positive control) and the purified RNA (negative control), using the three pairs of inter-gene primers listed in Supplementary Table S2. PCR products were visualized in a 1.4% agarose gel.

### Homology modeling or the response regulators

The protein sequences of the PsdR (locus *LCABL_16430*) and ApsR (locus *LCABL_19600*) response regulators were downloaded from Microbes online^49^. Protein sequence comparisons were performed with the online server BLAST Global Alignment with a Gap cost of: Existence:11, Extension:1 (Needleman-Wunsch Global Align Protein Sequences^50^). The amino acid sequences of the response regulators were used as input data into the Swiss Model which was used in the Automated Mode for the protein 3D structure predictions^30^. From the model results obtained for each sample, the one presenting a homodimer with the higher Global Model Quality Estimate (GMQE) after model building, and higher QMEANDisCo Global values, both ranging between 0 and 1, with higher numbers indicating higher expected accuracy of the resulting model, were used to create the final 3D-models presented here. For ApsR, the model based on the crystal structure of a hyperactive mutant of response regulator KdpE (a member of the OmpR/PhoB family) complexed to its promoter DNA (Template 4kfc.1.A^51^) was selected. This model presented a coverage of 0.98 with a sequence similarity of 0.35; the GMQE value was 0.69 and the QMEANDisCo Global was 0.67 ± 0.05. For PsdR, two homo-dimer models with the same GMQE value of 0.64, and the same QMEANDisCo Global value of 0.64 ± 0.05 were obtained based on (i) the crystal structure of a hyperactive mutant of response regulator KdpE complexed to its promoter DNA (Template 4kfc.1.A, X-ray, 2.53 Å^51^), and (ii) the crystal structure of *Klebsiella pneumoniae* PmrA (an OmpR/PhoB family response regulator) in complex with PmrA box DNA (Template 4s04.1.A; X-ray, 3.20 Å^52^). The model based on the crystal structure of the response regulator KdpE was selected for its higher resolution as recommended in the Swiss Model instructions for homology modeling. This model presented a coverage of 0.96 with a sequence similarity of 0.34.

### Expression and purification of His-tagged response regulators.

The coding regions of *psdR* and *apsR* were amplified by PCR using chromosomal DNA from *L. paracasei* BL23 as a template and primers RR09LIC-F and RR09LIC-R (for *psdR*) and RR12LIC-F and RR12LIC-R (for *apsR*) (Supplementary Table S2). The fragments were cloned into plasmid pNIC28-Bsa4 by ligase-independent cloning^53^. The resulting plasmids, pNICRR09 and pNICRR12, were used to transform *E. coli* BL21 (DE3) [pLysS], and the correct sequences of the inserts were confirmed by DNA sequencing. Bacterial cells were grown in 0.5 L of LB medium supplemented with chloramphenicol ((Sigma-Aldrich, Merck KGaA, Darmstadt, Germany)) and kanamycin ((Sigma-Aldrich, Merck KGaA, Darmstadt, Germany)) at 37 °C with agitation. When the cultures reached an OD_550_ of 0.5, 0.5 mM IPTG (isopropyl-β-d-thiogalactopyranoside, (Sigma-Aldrich, Merck KGaA, Darmstadt, Germany) was added and incubation was continued for 2 h.

For purification of PsdR and ApsR, cells were harvested by centrifugation, washed with 200 ml of cell resuspension buffer 1 (CRB1: 20 mM sodium phosphate [pH 7.4], NaCl 500 mM, imidazole 40 mM) and resuspended in 15 ml of CRB1. Cells were lysed in an Emulsiflex-B15 homogenizer (Avestin Inc.) by three passages at 2700 bar pressure. The cell debris was removed by centrifugation at 30,000×*g* for 20 min at 4°C. The cleared extracts were filtered through 0.22 µm pore size membranes and loaded onto HisTrap™ FF crude (1 ml) columns (GE Healthcare) equilibrated with CRB1. After the passage of the samples, the columns were washed with 10 ml of CRB1 and PsdR and ApsR were eluted with CRB1 supplemented with imidazole 2 M. Fractions were analyzed by SDS-PAGE, pooled and applied to Amicon® Ultra 15 mL Centrifugal Filters (10 kDa cutoff). The units were centrifuged at 4000×*g* for 20 min at 4°C and the buffer was replaced by Blitz buffer (BB; Tris:HCl 50 mM [pH 7.4], NaCl 150 mM, Tween 20 0.1% [v/v]) by three successive centrifugation steps adding 15 ml BB aliquots after each centrifugation. The final protein solutions were recovered and stored at −80 °C until use. Protein concentrations were determined with a Bio-Rad dye-binding assay.

### Biolayer interferometry assays (BLI)

The kinetic parameters of the interaction, binding affinity (K_D_), and rate constants of association (*k*_*a*_) and dissociation (*k*_*d*_) between PsdR or ApsR and DNA fragments encompassing predicted promoter binding sites (P_*psdA*_, P_*derA*_, P_*dlt*_ and P_*mprF*_; see Supplementary Table S3) or a non-specific DNA fragment used as a negative control (Flta), were measured by BLI using the BLITz system (FortéBio). To this purpose, 100 bp DNA fragments were synthesized by PCR using biotinylated reverse primers (Supplementary Table S2; Isogen Life Science), purified and dissolved in distilled water. The concentrations of the DNA fragments were measured by using a NanoDrop ND-1000 spectrophotometer (NanoDrop Technologies) and adjusted to 40 ng ml^−1^. Streptavidin biosensors (ForteBio) were equilibrated in BB for 30 min followed by the binding of the corresponding DNA fragment to the biosensor until saturation (15 min). Before the assays, His-tagged ApsR or PsdR were incubated in BB buffer supplemented with acetyl-phosphate (50 mM) for ApsR or ammonium phosphoramidate (50 mM) for PsdR, and MgCl_2_ (100 mM) for 30 min and conveniently diluted with BB. For each interaction, five dilutions of the protein plus a reference without protein added were assayed. Kinetics values calculation and data analysis were performed with BLItz Pro 1.2 software using a 1:1 stoichiometry model.

### Supplementary Information

Supplementary Information.

## Data Availability

The raw data supporting the conclusions of this article will be made available by the authors, without undue reservation, to any qualified researcher.

## References

[1] Stock AM, Robinson VL, Goudreau PN (2000). Two-component signal transduction. Annu. Rev. Biochem..

[2] Capra EJ, Laub MT (2012). Evolution of Two-component signal transduction systems. Annu. Rev. Microbiol..

[3] Gao R, Stock AM (2009). Biological insights from structures of Two-component proteins. Annu. Rev. Microbiol..

[4] Laub MT, Goulian M (2007). Specificity in Two-component signal transduction pathways. Annu. Rev. Genet..

[5] Zschiedrich CP, Keidel V, Szurmant H (2016). Molecular mechanisms of Two-component signal transduction. J. Mol. Biol..

[6] Mascher T, Margulis NG, Wang T, Ye RW, Helmann JD (2003). Cell wall stress responses in *Bacillus subtilis*: The regulatory network of the bacitracin stimulon. Mol. Microbiol..

[7] Ohki R (2003). The BceRS Two-component regulatory system induces expression of the bacitracin transporter, BceAB, *Bacillus subtilis*. Mol. Microbiol..

[8] Mascher T (2014). Bacterial (intramembrane-sensing) histidine kinases: Signal transfer rather than stimulus perception. Trends Microbiol..

[9] Dintner S (2011). Coevolution of ABC transporters and Two-component regulatory systems as resistance modules against antimicrobial peptides in *Firmicutes* Bacteria. J. Bacteriol..

[10] Dintner S, Heermann R, Fang C, Jung K, Gebhard S (2014). A sensory complex consisting of an ATP-binding cassette transporter and a two-component regulatory system controls bacitracin resistance in *Bacillus subtilis*. J. Biol. Chem..

[11] Joseph P, Fichant G, Quentin Y, Denizot F (2002). Regulatory relationship of Two-component and ABC transport systems and clustering of their genes in the Bacillus/Clostridium group, suggest a functional link between them. J. Mol. Microbiol. Biotechnol..

[12] Revilla-Guarinos A, Gebhard S, Mascher T, Zúñiga M (2014). Defence against antimicrobial peptides: Different strategies in Firmicutes. Environ. Microbiol..

[13] Koh A, Gibbon MJ, Van der Kamp MW, Pudney CR, Gebhard S (2021). Conformation control of the histidine kinase BceS of *Bacillus subtilis* by its cognate ABC-transporter facilitates need-based activation of antibiotic resistance. Mol. Microbiol..

[14] Fritz G (2015). A new way of sensing: Need-based activation of antibiotic resistance by a flux-sensing mechanism. mBio.

[15] Alcántara C, Revilla-Guarinos A, Zúñiga M (2011). Influence of Two-component signal transduction systems of *Lactobacillus casei* BL23 on tolerance to stress conditions. Appl. Environ. Microbiol..

[16] Monedero, V., Revilla-Guarinos, A. & Zúñiga, M. in *Advances in applied microbiology* Vol. 99 Ch. One, Physiological Role of Two-Component Signal Transduction Systems in Food-Associated Lactic Acid Bacteria., 1–51 (Academic Press, 2017). 10.1016/bs.aambs.2016.12.002 28438266

[17] Revilla-Guarinos A (2013). Characterization of a regulatory network of peptide antibiotic detoxification modules in *Lactobacillus casei* BL23. Appl. Environ. Microbiol..

[18] Revilla-Guarinos A (2020). ABC transporter DerAB of *Lactobacillus casei* mediates resistance against insect-derived defensins. Appl. Environ. Microbiol..

[19] Neuhaus FC, Baddiley J (2003). A continuum of anionic charge: Structures and functions of D-alanyl-teichoic acids in gram-positive bacteria. Microbiol. Mol. Biol. Rev..

[20] Weidenmaier C, Peschel A (2008). Teichoic acids and related cell-wall glycopolymers in Gram-positive physiology and host interactions. Nat. Rev. Microbiol..

[21] Swoboda JG, Campbell J, Meredith TC, Walker S (2010). Wall teichoic acid function, biosynthesis, and inhibition. Chembiochem.

[22] Peschel A (2001). *Staphylococcus aureus* resistance to human defensins and evasion of neutrophil killing via the novel virulence factor MprF is based on modification of membrane lipids with l-lysine. J. Exp. Med..

[23] Oku Y, Kurokawa K, Ichihashi N, Sekimizu K (2004). Characterization of the *Staphylococcus aureus mprF* gene, involved in lysinylation of phosphatidylglycerol. Microbiology (Reading).

[24] Andrä J, Goldmann T, Ernst CM, Peschel A, Gutsmann T (2011). Multiple peptide resistance factor (MprF)-mediated Resistance of *Staphylococcus aureus* against antimicrobial peptides coincides with a modulated peptide interaction with artificial membranes comprising lysyl-phosphatidylglycerol. J. Biol. Chem..

[25] Ernst CM, Peschel A (2011). Broad-spectrum antimicrobial peptide resistance by MprF-mediated aminoacylation and flipping of phospholipids. Mol. Microbiol..

[26] Ernst CM (2009). The bacterial defensin resistance protein MprF consists of separable domains for lipid lysinylation and antimicrobial peptide repulsion. PLoS Pathog..

[27] Thedieck K (2006). The MprF protein is required for lysinylation of phospholipids in listerial membranes and confers resistance to cationic antimicrobial peptides (CAMPs) on *Listeria monocytogenes*. Mol. Microbiol..

[28] Revilla-Guarinos A, Alcántara C, Rozès N, Voigt B, Zúñiga M (2014). Characterization of the response to low pH of Lactobacillus casei ΔRR12, a mutant strain with low D-alanylation activity and sensitivity to low pH. J. Appl. Microbiol..

[29] Capra EJ, Perchuk BS, Skerker JM, Laub MT (2012). Adaptive mutations that prevent crosstalk enable the expansion of paralogous signaling protein families. Cell.

[30] Waterhouse A (2018). SWISS-MODEL: Homology modelling of protein structures and complexes. Nucleic Acids Res..

[31] Bourret RB (2010). Receiver domain structure and function in response regulator proteins. Curr. Opin. Microbiol..

[32] Castro NSS (2018). Small phospho-donors phosphorylate MorR without inducing protein conformational changes. Biophys. Chem..

[33] Rietkötter E, Hoyer D, Mascher T (2008). Bacitracin sensing in *Bacillus subtilis*. Mol. Microbiol..

[34] Darwin AJ, Tyson KL, Busby SJ, Stewart V (1997). Differential regulation by the homologous response regulators NarL and NarP of *Escherichia coli* K-12 depends on DNA binding site arrangement. Mol. Microbiol..

[35] Rajeev L (2011). Systematic mapping of Two component response regulators to gene targets in a model sulfate reducing bacterium. Genome Biol..

[36] Bielecki P (2015). Cross talk between the response regulators PhoB and TctD allows for the integration of diverse environmental signals in *Pseudomonas aeruginosa*. Nucleic acids Res..

[37] Randall CP (2018). Acquired nisin resistance in *Staphylococcus aureus* involves constitutive activation of an intrinsic peptide antibiotic detoxification module. mSphere.

[38] Blake KL, Randall CP, O'Neill AJ (2011). *In vitro* studies indicate a high resistance potential for the lantibiotic nisin in *Staphylococcus aureus* and define a genetic basis for nisin resistance. Antimicrob Agents Chemother.

[39] Draper LA, Cotter PD, Hill C, Ross RP (2015). Lantibiotic resistance. Microbiol. Mol. Biol. Rev..

[40] Jaumaux F, P. Gómez de Cadiñanos L, Gabant P (2020). In the age of Synthetic Biology, will antimicrobial peptides be the next generation of antibiotics?. Antibiotics (Basel).

[41] Prater AG (2019). Environment shapes the accessible daptomycin resistance mechanisms in *Enterococcus faecium*. Antimicrob Agents Chemother.

[42] Prater AG (2021). Daptomycin resistance in *Enterococcus faecium* can be delayed by disruption of the LiaFSR stress response pathway. Antimicrob Agents Chemother.

[43] Morris SM, Fritz G, Rogers T, Gebhard S (2022). Novel regulatory logic in the antibiotic resistance response of *Enterococcus faecalis* against cell envelope targeting antibiotics. bioRxiv.

[44] Zheng J (2020). A taxonomic note on the genus *Lactobacillus*: Description of 23 novel genera, emended description of the genus *Lactobacillus* Beijerinck 1901, and union of *Lactobacillaceae* and *Leuconostocaceae*. Int. J. Syst. Evol. Microbiol..

[45] Banerjee S, Hansen JN (1988). Structure and expression of a gene encoding the precursor of subtilin, a small protein antibiotic. J. Biol. Chem..

[46] Sambrook J, Fritsch EF, Maniatis T (1989). Molecular cloning: A laboratory manual.

[47] Posno M (1991). Incompatibility of *Lactobacillus* vectors with replicons derived from small cryptic *Lactobacillus* plasmids and segregational instability of the introduced vectors. Appl. Environ. Microbiol..

[48] Landete JM (2010). Requirement of the *Lactobacillus casei* MaeKR two-component system for L-malic acid utilization via a malic enzyme pathway. Appl. Environ. Microbiol..

[49] Dehal PS (2010). MicrobesOnline: An integrated portal for comparative and functional genomics. Nucleic Acids Res..

[50] Altschul SF (1997). Gapped BLAST and PSI-BLAST: A new generation of protein database search programs. Nucleic Acids Res..

[51] Narayanan A, Kumar S, Evrard AN, Paul LN, Yernool DA (2014). An asymmetric heterodomain interface stabilizes a response regulator-DNA complex. Nat. Commun..

[52] Lou YC (2015). Structure and dynamics of polymyxin-resistance-associated response regulator PmrA in complex with promoter DNA. Nat. Commun..

[53] Savitsky P (2010). High-throughput production of human proteins for crystallization: The SGC experience. J. Struct. Biol..

