# Supplementary material for: Cross-regulation of Aps-promoters in Lacticaseibacillus paracasei by the PsdR response regulator in response to lantibiotics
Source: Sci Rep. 2024 Feb 9;14:3319. doi: 10.1038/s41598-024-53592-1 (PMC10858260; doi:10.1038/s41598-024-53592-1)
Supplement: Supplementary file 1 — Supplementary Information. [file 41598_2024_53592_MOESM1_ESM.pdf]

SUPPLEMENTARY MATERIAL

Cross-regulation of Aps-promoters in *Lacticaseibacillus paracasei* by the PsdR response regulator in response to lantibiotics

Qian Zhang [1], Manuel Zúñiga [2], Cristina Alcántara [2], Diana Wolf [1], Thorsten Mascher [1*], and Ainhoa Revilla-Guarinos [1*¥]

[1] Chair of General Microbiology, Technische Universität Dresden, 01217 Dresden, Germany; qian.zhangwu1991@gmail.com, thorsten.mascher@tu-dresden.de, diana.wolf1@tu-dresden.de

[2] Departamento de Biotecnología, Instituto de Agroquímica y Tecnología de Alimentos (IATA), Consejo Superior de Investigaciones Científicas (CSIC), 46980 Paterna, Valencia, Spain; btcman@iata.csic.es (M. Z.), btccab@iata.csic.es (C. A.).

[¥] Present address A. R-G.: Oral Microbiome Group, Genomics and Health Department, FISABIO Foundation, 46020 Valencia, Spain; ainhoa.revilla@fisabio.es.

*****Correspondence: ainhoa.revilla@fisabio.es, thorsten.mascher@tu-dresden.de

**Supplementary material**	**Content**
Supplementary Table S1	Strains and plasmids used in this study
Supplementary Table S2	Primers used in this study
Supplementary Table S3	Fragments used for interferometry assays
Supplementary Figure S1	Sensitivity assays of *L. paracasei* BL23 and *ΔdltA* towards subtilin
Supplementary Figure S2	Killing assays for *ΔpsdRΔapsR* with subtilin
Supplementary Figure S3	Analysis of the phosphorylation of PsdR and ApsR with acetyl-phosphate or ammonium phosphoramidate
Supplementary Figure S4	Original gel for the RT-PCR analysis of the *psdRSAB* operon transcripts
Supplementary References	

Supplementary Table S1. Strains and plasmids used in this study.

Strains/ plasmid	Description[a]	Source /Reference
Lacticaseibacillus paracasei strains		
L. paracasei BL23	Wild type	B. Chassy, U. Illinois
L. paracasei ΔderB	BL23 ΔLCABL_21670	[1]
L. paracasei ΔdltA	LCABL_08550 (*dltA*)::pRV08550, Ery^r	[2]
L. paracasei ΔmprF	LCABL_24490 (*mprF*):: pRV24490, Ery^r	[2]
L. paracasei ΔpsdB	BL23 ΔLCABL_16400	This study
L. paracasei ΔpsdR	BL23 ΔLCABL_16430	[2]
L. paracasei ΔapsB	LCABL_19580 *(apsB)*::pRV19580, Ery^r	[2]
L. paracasei ΔapsR	BL23 ΔLCABL_19600	[3]
L. paracasei ΔpsdR ΔapsR	BL23 ΔLCABL_16430; LCABL_19600 *(apsR)*::pRV19600, Ery^r	This study
L. paracasei ΔderB ΔpsdB	BL23 ΔLCABL_21670 ΔLCABL_16400	This study
L. paracasei ΔderB ΔpsdR	BL23 ΔLCABL_21670; LCABL_16430 (*psdR)*::pRV16430, Ery^r	This study
L. paracasei ΔderB ΔapsB	BL23 ΔLCABL_21670; LCABL_19580 (*apsB)*::pRV19580, Ery^r	This study
L. paracasei ΔderB ΔapsR	BL23 ΔLCABL_21670; LCABL_19600 *(apsR)*::pRV19600, Ery^r	This study
L. paracasei ΔpsdB ΔdltA	BL23 ΔLCABL_16400; LCABL_08550 (*dltA*)::pRV08550, Ery^r	This study
L. paracasei ΔpsdR ΔdltA	BL23 ΔLCABL_16430; LCABL_08550 (*dltA*)::pRV08550, Ery^r	This study
L. paracasei ΔderB ΔdltA	BL23 ΔLCABL_21670; LCABL_08550 *(dltA)::*pRV08550*, Ery^r*	[1]
L. paracasei ΔderB ΔpsdB ΔdltA	BL23 ΔLCABL_21670 ΔLCABL_16400; LCABL_08550 *(dltA)::*pRV08550, Ery^r	This study
**Other strains**		
Escherichia coli DH10β	F$^-$ *mcrA* Δ(*mrr-hsdRMS-mcrBC*) Φ80d*lacZΔM15* Δ*lacX74 endA1 recA1 deoR* Δ(*ara,leu*)7697 *araD139 galU galK nupG rpsL* λ$^-$	Stratagene
Escherichia coli BL21 (DE3) [pLysS]	F$^-$ *ompT gal dcm lon* $hsdS_B(r_B^-\ m_B^-)$ λ(DE3) pLysS(Chl^r)	Novagen
Bacillus subtilis ATCC 6633	subtilin producer	Laboratory stock
**Vectors and Plasmids**		
pRV300	Insertional vector for *Lactobacillus*, Amp^r, Ery^r	[4]
pRV300-*psdB-del*	pRV300 containing fused up- and down-flanking fragments of LCABL_16400 to generate clean deletion of *psdB*; Amp^r, Ery^r	This study
pRV19600	pRV300 containing a 382-pb internal fragment of LCABL_19600 (*apsR*), Amp^r, Ery^r	[3]
pRV19580	pRV300 containing a 773-pb internal fragment of LCABL_19580 (*apsB*), Amp^r, Ery^r	[2]
pRV16430	pRV300 containing a 354-pb internal fragment of LCABL_16430 (*psdR*), Amp^r, Ery^r	[3]
pRV08550	pRV300 containing a 679-pb internal fragment of LCABL_08550 (*dltA*), Amp^r, Ery^r	[2]
pNIC28-Bsa4	*cis*-repressed, IPTG-inducible, N-terminal His6-tagged recombinant protein overexpression vector; Kan^r	[5]
pNIC-RR09	pNIC28-Bsa4 with cloned *psdR* gene; Kan^r	This study
pNIC-RR12	pNIC28-Bsa4 with cloned *apsR* gene; Kan^r	This study

[a]Amp^r: ampicillin resistance; Ery^r: erythromycin resistance; Kan^r: kanamycin resistance; Chl^r: chloramphenicol resistance.

Supplementary Table S2. Primers used in this study

Primer number	Primer descriptive name or reference	Primer sequence[a] 5'→3'	Application
**Cloning of *L. paracasei* mutant strains**			
RG001	pRV300.Fw	GTTTTCCCAGTCACGAC	Vector-binding primer
RG002	pRV300.Rv	CAGGAAACAGCTATGAC	Vector-binding primer
RG003	Lsei.1738.Rv2	GTCCGATCACTGACAAGC	checking insertional inactivation of *apsB*
RG019	Lsei_dltA.Fw1.RT-PCR	TGGTCGAGGTTTTCTTGGGC	checking insertional inactivation of *dltA*
RG212	UpFw-Lca-psdB	TTTT**GAATTC**TAAGCTACCTTACCAGCTT	*psdB* clean deletion
RG213	UpRv-Lca-psdB	CTGGCGTTTCGACGCTTTTTTTAGTTATT TCATTGTCCATCGCCTGCCTTTG	*psdB* clean deletion
RG214	doFw-Lca-psdB	CAAAGGCAGGCGATGGACAATGAAATA ACTAAAAAAAGCGTCGAAACGCCAG	*psdB* clean deletion
RG215	doRv-Lca-psdB	AAAA**GAGCTC**ATCGGCATACGGTCCAAG TTGTTC	*psdB* clean deletion
RG149	Check-mod9_5	CAAACGATCCTCATGGTAAC	Checking construct pRG300-*psdB*-del
RG083	Permease9ATPase9.rv	AATTTTGTTGGCTGATGAACCC	Checking construct pRG300-*psdB*-del
RG183	ABC9ret-R	CTAACTGTCACCTCAGAAATTG	Checking *psdB* clean deletion
RG216	xerC-fw	CAACAACTGCCAGCCAAGCC	Checking *psdB* clean deletion
C92-59224	[3]	CCTAAAAGCCGGACAAACCC	checking insertional inactivation of *apsR*
C83-40666	[3]	CAGTTAGAATAGAAACTGTCC	checking insertional inactivation of *psdR*
**qRT-PCR**			
RG010	lepA-F	CACATTGATCACGGGAAGTC	Reference gene
RG011	lepA-R	GTAATGCCACGTTCACGTTC	Reference gene
RG012	ileS-F	ACCATTCCGGCTAACTATGG	Reference gene
RG013	ileS-R	TCAGGATCTTCGGATTTTCC	Reference gene
RG014	pcrA-F	CGGCCAATAATGTGATTCAG	Reference gene
RG015	pcrA-R	TCATCAGTTTCGCTTTGAGC	Reference gene
RG016	pyrG-F	AATTGCGCTTTTCACTGATG	Reference gene
RG017	pyrG-R	CGAAATGATCGACCACAATC	Reference gene
RG006	Lsei_1738.Fw3.RT-PCR	GGGAACGCGCATTCATTGTG	Amplification of *apsB* region
RG007	Lsei_1738.Rv3.RT-PCR	TCTCGCGCTGAACAAGATCC	Amplification of *apsB* region
RG008	Lsei_1993.Fw2.RT-PCR	TTGCCGGTATTTTGGTCGGG	Amplification of *derB* region
RG009	Lsei_1993.Rv2.RT-PCR	ATGTCCACAATACGGCTGGC	Amplification of *derB* region
RG019	Lsei_dltA.Fw1.RT-PCR	TGGTCGAGGTTTTCTTGGGC	Amplification of *dltA* region
RG020	Lsei_dltA.Rv1.RT-PCR	CCGGTGTATGGGCAACATCC	Amplification of *dltA* region
RG021	Lsei_mprF.Fw1.RT-PCR	GCCGGATCAGCCAAGACTTG	Amplification of *mprF* region
RG022	Lsei_mprF.Rv1.RT-PCR	TTAGCATCGGTGTAACGGCG	Amplification of *mprF* region
RG027	ATPasa-12.Fw1.RT-PCR	TAGCTTTCAGGTCAACGCGG	Amplification of *apsA* region
RG028	ATPasa-12.Rv1.RT-PCR	CTTGGCGTCTCAATCGTTGC	Amplification of *apsA* region
RG029	RR12. Fw1.RT-PCR	GGCAATGAATATGGGCGCTG	Amplification of *apsR* region

RG030	RR12. Rv1.RT-PCR	TAGGTTCGTCGAAGCAAGGC	Amplification of *apsR* region
RG031	ATPasa-1994.Fw1.RT-PCR	CACCCGCATTGAAAGGTGTC	Amplification of *derA* region
RG032	ATPasa-1994.Rv1.RT-PCR	GCAAGGTCGTTTTCCCTGAAC	Amplification of *derA* region
RG033	ATPasa-9.Fw1.RT-PCR	GGACAGGATCTGAGCAACGTC	Amplification of *psdA* region
RG034	ATPasa-9.Rv1.RT-PCR	ATTGAAGGTGTCAAGCAAGTCG	Amplification of *psdA* region
RG054	Lsei_1417.Fw3.RT-PCR	GTACCGTCCTTTCCCGCATC	Amplification of *psdB* region
RG055	Lsei_1417.Rv3.RT-PCR	CCGATGGTAATGATCCCGGC	Amplification of *psdB* region
RG056	RR9.Fw2.RT-PCR	AGCGAGTTACGCAAACACAG	Amplification of *psdR* region
RG057	RR9.Rv2.RT-PCR	CGGCTCCTAAGTTCATCGCC	Amplification of *psdR* region
**RT-PCR to detect polycistronic transcripts**			
RG078	HK9-RR9.fw	AGCTTTTGCGTCAGCGTTTG	Amplification of intergenic region *psdS-psdR*
RG079	HK9-RR9.rv	GGCGATGAACTTAGGAGCCG	
RG080	ATPase9-HK9.fw	AGCAGATCAAGCAGTTCTGTAG	Amplification of intergenic region *psdA-psdS*
RG081	ATPase9-HK9.rv	AGCATTGATTATTGGCGCTTTC	
RG082	Permease9-ATPase9.fw	TCGCAACCATTTTAAGACGAAG	Amplification of intergenic region *psdB-psdA*
RG083	Permease9-ATPase9.rv	AATTTTGTTGGCTGATGAACCC	
**Protein expression**			
	RR09LIC-F	TACTTCCAATCCATGGCACAGAAAATTTTTATTGTCG	Cloning of *psdR*
	RR09LIC-R	TATCCACCTTTACTGCTATTATCATGGCTTTGGTCCCTCAC	Cloning of *psdR*
	RR12LIC-F	TACTTCCAATCCATGTTTAAAATCATGAT	Cloning of *apsR*
	RR12LIC-R	TATCCACCTTTACTGTCATTACTAAGGAACGATGTAACCTTG	Cloning of *apsR*
**BLI assays**			
	P09-F	GTAAGCTACCTTACCAGCTTG	P_{psdA} fragment
	P09-R	CATGTGATCGTCCCCTTTC	P_{psdA} fragment
	Por-F	GGTAAAGAAAGACTAAAGCAGC	P_{derA} fragment
	Por-R	CTTTATGATAGCGGGTTAGGC	P_{derA} fragment
	Pdlt-F	TCACGCTTCTCATATTACGA	P_{dltA} fragment
	Pdlt-R	CCACGTTTTTATTTACACATTCA	P_{dltA} fragment
	Pmpr-F	CAATCATCTCGCAAACTTACATC	P_{mprF} fragment
	Pmpr-R	GTCCTCCGTGTCTATATTCC	P_{mprF} fragment
	Flta-F	AAACTGCCGATGCTGAG	Flta fragment
	Flta-R	GCTGCCTGGAAAGTATTGG	Flta fragment

[a] Characters in bold: recognition sites of restriction enzymes.

Supplementary Table S3. Fragments used for interferometry assays

**Fragment name**	**Sequence 5'→3'**	**Coordinates in *L. casei* BL23 genome sequence (Acc. Nº FM177140)**
P_{psdA} (promoter region upstream of *psdA*)	GTAAGCTA***CCTTACC***AGCT***TGTAAGC***AACTACGAATCACTGTAAGTAGGCATGGGGCATCATCCCCGCTAAGCTAATAAATGA**AAGGGG**ACGATCACATG[1]	1581782-1581683
P_{derA} (promoter region upstream of *derA*)	GGTAAAGAAAGACTAAAGCA***GCTTACA***AAGC***CGTAAGT***TACAAACAACCATCCGTTAGGCCATTATCAGGAAAAAGGCAGCCTAACCCGCTATCATAAAG[2]	2096912-2096813
P_{dltA} (promoter region upstream of *dltA*)	TCACGCTTCTCA***TATTACG***AAAC***TGTTAGC***TCATTGTTACGCAATTCGTTGGCGTCATTTTTTCTGAAAACCTATAATGAATGTGTAAATAAAAACGTGG[2]	851054-851153
P_{mprF} (promoter region upstream of *mprF*)	CAATCATCTCGCAAACTTACATCA***CCTTACA***AAAA***GGTCAGA***AAGGGTGCGAGGGGCTTGTAGATTTCCTATAATGAATAGGAATATAGACACGGAGGAC[2]	2403722-2403623
Flta (fragment within *yfnI* coding sequence)	AAACTGCCGATGCTGAGATGATGATGGAAAACTCACTGTTTGGTTTACCTGAAGGGGCAGCCATGGTTACCGATGGCACAACCAATACTTTCCAGGCAGC	905844-905884

[1] Stop and start translational sites of *psdS* and *psdA*, respectively, are underlined. Putative Shine-Dalgarno box is underlined bold characters. The PsdR putative binding site is indicated in italics bold characters.
[2] The ApsR putative binding site is indicated in italics bold characters.

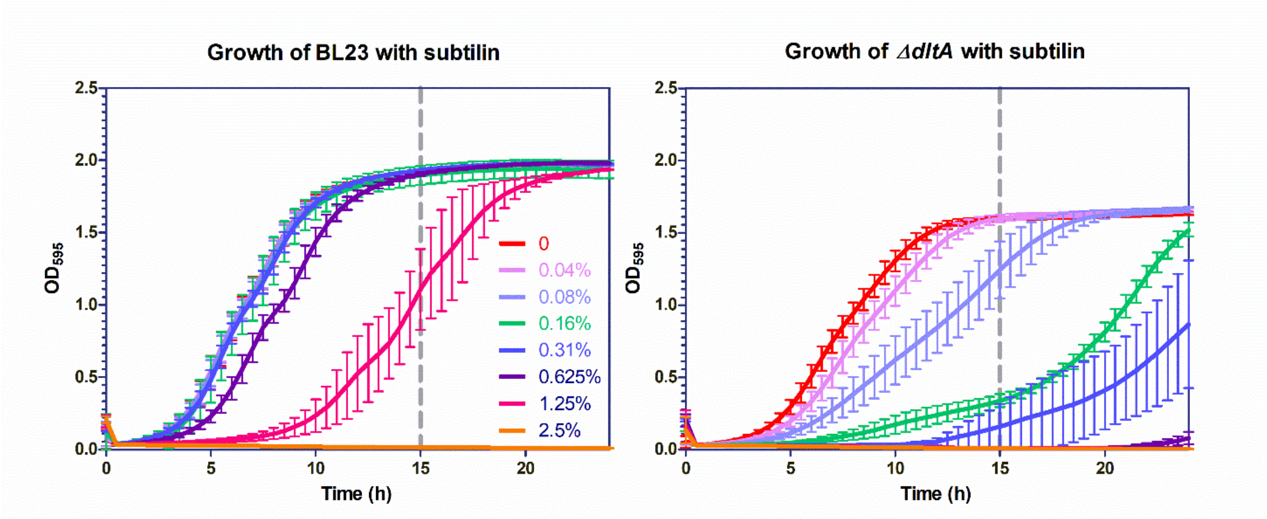

Supplementary Figure S1. Sensitivity assays of *L. paracasei* BL23 and *ΔdltA* towards subtilin. The susceptibility of *L. paracasei* BL23 and *ΔdltA* to subtilin was examined by treating the strains with a series of dilutions of subtilin-containing supernatant. Cells from overnight cultures were harvested by centrifugation and washed twice using two volumes of 0.1 % (w/v) peptone-water. After the last washing, the cell pellet was resuspended in MRS to a final $OD_{595} \approx 0.1$. The cells were dispensed in 96 well plates to a final OD_{595} of 0.05, and serial-dilutions of subtilin in MRS were added to reach the desired final concentrations. MRS media was added to the reference —untreated— cultures. Growth at 37 °C without shaking was monitored for 24 h using a Synergy™ NEO multi-mode microplate reader from BioTek® (Winooski, VT, USA). The MIC_{15H} was defined as the lowest concentration of antibiotic that completely inhibited bacterial growth at 15 hours (indicated by a grey vertical dashed line). Subtilin concentrations are indicated in the BL23 graph. The experiments were performed at least in triplicate. Means and SDs are depicted. The same procedure was performed with the rest of the mutant strains.

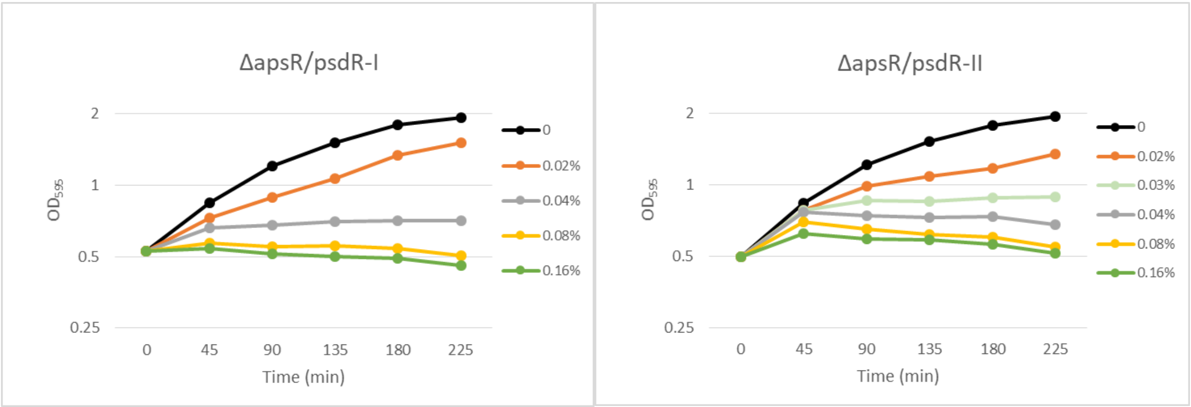

Supplementary Figure S2. Killing assays for *ΔpsdRΔapsR* with subtilin.

The subtilin concentration used for the transcriptomic studies was determined by exposing mid-exponential growing cultures ($OD_{595} \approx 0.5$) to a series of subtilin dilutions, with one untreated control sample. The effect on growth was determined by measuring the OD_{595} every 45 min for more than 3 hours. Selection of the subinhibitory concentration of subtilin was based on the phenotype of the most sensitive mutant *ΔpsdRΔapsR*, so that addition of subtilin had a significant effect on the growth rate of the culture without completely inhibiting it. Two replicas are shown. The subtilin concentrations used are indicated in the graphs.

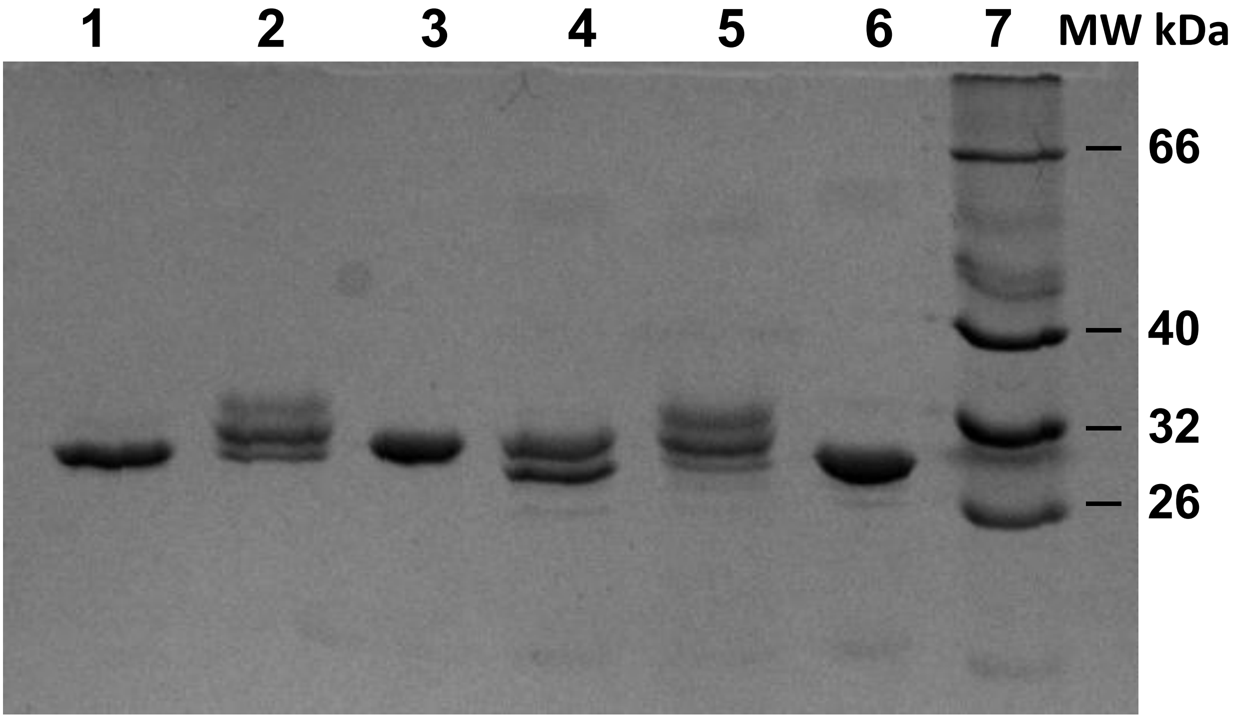

Supplementary Figure S3. Analysis of the phosphorylation of PsdR and ApsR with acetyl-phosphate or ammonium phosphoramidate in 10% SDS-PAGE gel stained with Coomasie blue. His-tagged ApsR or PsdR were incubated in BB buffer supplemented with acetyl-phosphate (50 mM) or ammonium phosphoramidate (50 mM), and $MgCl_2$ (100 mM) for 30 minutes and conveniently diluted with BB. From left to right: 1) PsdR with Acetyl-phosphate, 2) PsdR with phosphoramidate, 3) PsdR not phosphorylated, 4) ApsR phosphorylated with Acetyl-phosphate, 5) ApsR phosphorylated with phosphoramidate, 6) ApsR not phosphorylated, 7) molecular weight ladder.

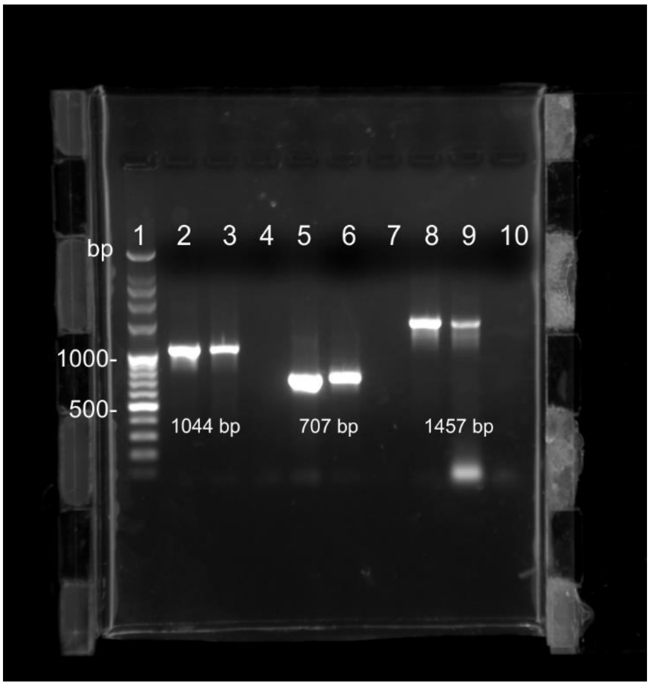

Supplementary Figure S4. Original gel for the RT-PCR analysis of the *psdRSAB* operon transcripts.
The PCR amplification products correspond to the following cross-gene primers: RG082 – RG083 (lines 2-4, amplification of intergenic region psdB-psdA, product size [1044 bp]); RG078 – RG079 (lines 5-7, amplification of intergenic region psdS-psdR [707 bp]); and RG080 – RG081 (lines 8-10; amplification of intergenic region psdA-psdS [1457 bp]); line 1 contains the molecular weight ladder. Negative controls using RNA as template are presented in lines 4, 7 and 10 to verify the absence of residual genomic DNA in the purified RNA samples. Positive controls amplified from genomic DNA are presented in lines 2, 5 and 8. Lines 3, 6 and 9 contain the RT-PCR products amplified from the cDNA.

References for the Supplementary materials:

1 Revilla-Guarinos, A. *et al.* ABC transporter DerAB of *Lactobacillus casei* mediates resistance against insect-derived defensins. *Applied and environmental microbiology* **86,** AEM.00818-00820, doi:10.1128/AEM.00818-20 (2020).

2 Revilla-Guarinos, A. *et al.* Characterization of a regulatory network of peptide antibiotic detoxification modules in *Lactobacillus casei* BL23. *Appl. Environ. Microbiol.*, doi:10.1128/AEM.00178-13 (2013).

3 Alcántara, C., Revilla-Guarinos, A. & Zúñiga, M. Influence of Two-component signal transduction systems of *Lactobacillus casei* BL23 on tolerance to stress conditions. *Appl. Environ. Microbiol.* **77,** 1516-1519, doi:10.1128/aem.02176-10 (2011).

4 Leloup, L., Ehrlich, S. D., Zagorec, M. & Morel-Deville, F. Single-crossover integration in the *Lactobacillus sake* chromosome and insertional inactivation of the *ptsI* and *lacL* genes. *Appl. Environ. Microbiol.* **63,** 2117-2123 (1997).

5 Savitsky, P. *et al.* High-throughput production of human proteins for crystallization: The SGC experience. *Journal of structural biology* **172,** 3-13, doi:https://doi.org/10.1016/j.jsb.2010.06.008 (2010).
